# A Meta-Analysis Reveals the Commonalities and Differences in *Arabidopsis thaliana* Response to Different Viral Pathogens

**DOI:** 10.1371/journal.pone.0040526

**Published:** 2012-07-12

**Authors:** Guillermo Rodrigo, Javier Carrera, Virgina Ruiz-Ferrer, Francisco J. del Toro, César Llave, Olivier Voinnet, Santiago F. Elena

**Affiliations:** 1 Instituto de Biología Molecular y Celular de Plantas, Consejo Superior de Investigaciones Científicas - Universidad Politécnica de Valencia, València, Spain; 2 Instituto ITACA, Universidad Politécnica de Valencia, València, Spain; 3 Institut de Biologie Moléculaire des Plantes, CNRS, Strasbourg, France; 4 Centro de Investigaciones Biológicas, CSIC, Madrid, Spain; 5 Santa Fe Institute, Santa Fe, New Mexico, United States of America; United States Department of Agriculture, Agricultural Research Service, United States of America

## Abstract

Understanding the mechanisms by which plants trigger host defenses in response to viruses has been a challenging problem owing to the multiplicity of factors and complexity of interactions involved. The advent of genomic techniques, however, has opened the possibility to grasp a global picture of the interaction. Here, we used *Arabidopsis thaliana* to identify and compare genes that are differentially regulated upon infection with seven distinct (+)ssRNA and one ssDNA plant viruses. In the first approach, we established lists of genes differentially affected by each virus and compared their involvement in biological functions and metabolic processes. We found that phylogenetically related viruses significantly alter the expression of similar genes and that viruses naturally infecting *Brassicaceae* display a greater overlap in the plant response. In the second approach, virus-regulated genes were contextualized using models of transcriptional and protein-protein interaction networks of *A. thaliana*. Our results confirm that host cells undergo significant reprogramming of their transcriptome during infection, which is possibly a central requirement for the mounting of host defenses. We uncovered a general mode of action in which perturbations preferentially affect genes that are highly connected, central and organized in modules.

## Introduction

For decades, plant molecular virology has been overly focused on the pathogen itself, studying their individual genes and products, and their local effects on certain regulatory pathways related to antiviral responses. However, with the arrival of modern genomic tools allowing for high-throughput screenings, we can now tackle the problem of the plant host-virus interaction from a systemic perspective that would allow us reaching a deeper understanding on how host and virus genotypes, environmental effects and stochasticity interplay in determining the pathological outcome of an infection. Viral infections typically alter host physiology, notably by diverting almost all cellular resources for the production of virus-specific components, and by actively suppressing host defenses [1], [2]. As a response to infection, hosts compensate by over- or under-expressing certain cellular pathways, and deploying specific antiviral measures. Collectively, these alterations determine the type and strength of symptoms displayed and the virulence of the infection. Much effort has gone into identifying individual cellular traits that may change as a consequence of viral infection [3] and this has greatly benefited from the contemporary development of genome-wide investigation technologies and their successful application to plant diseases research [4], [5]. These technologies have further demonstrated great potential in providing insights into multidimensional networks of plant-virus interactions [4], [6], notably by allowing combined analyses at the host transcriptome and proteome levels, as was recently shown for HIV-1 [7].

Based on the above, it has been anticipated that a systems biology approach to infections should allow the identification of universal principles and features of host-virus interactions, as opposed to scrutinizing many specific aspects of any given viral infection [8]–[10]. Such generic principles may indeed prove more predictive of the outcome of viral diseases and therefore, more efficient in the prophylaxis, diagnosis, and even treatment of such diseases. In a network approach, viral pathogenesis can be viewed as the expression of new constraints imposed by the virus upon the cellular interactome: while the host initiates a reprogramming of its genetic profile to activate the immune system to counteract the infection effects, replication and suppression of host defenses by viruses entail the manipulation of molecular connections that ultimately result in the misregulation and/or silencing of genes that trigger defense functions, and eventually in the emergence of new topological properties of the host interactome. Thus, understanding the bases for such modifications is crucial to acquire a systemic view of the infection process [1], [11], [12]. One of the main goals to this end would be the identification of the host proteins interacting with the virus (i.e., the targets of the viral proteins). Instead, herein we focus on the study of the mechanisms by which the host canalizes these virus targets to trigger the global defense system. We propose a reverse-engineering approach by which we analyze the genetic profile of the cell upon viral infection and contextualize this information onto the host interaction network.

Analyses of interaction networks have already uncovered global, dynamic features that relate directly to biological properties [13]. For example, proteins with a large number of interactions within a network, also referred to as ‘hubs’, have a higher impact on multiple phenotypic traits (pleiotropy) than loosely connected proteins. Moreover, proteins essential for survival are highly clustered [14]. Hub proteins can be further partitioned into those that function in a specific biological module and those that connect different modules. The existence of such hub proteins generates two interesting properties in networks. First, the network is scale-free in that the number of connections per node (i.e., its connectivity or degree) probability distribution follows asymptotically a power-law. Second, the network presents the characteristic of small-worlds, in which the average number of intermediary nodes connecting any random pair is small [15]. These two properties confer robustness against random perturbations in the network, but at the cost of strong sensitivity to attacks directed against hubs [16]. Although plant viruses usually encode for few proteins, the genetic profile of the host after viral infection presents hundreds and even thousands of significant changes. A plausible explanation for this scenario is that the host proteins interacting with the virus are highly connected nodes that spread the signal, and additionally interact in a short downstream pathway with the immune response genes. Of relevance, very recently it has been experimentally shown that bacterial effector targets in *Arabidopsis thaliana* are hubs and canalize the signal onto the regulators of the global immune system [17]. Interestingly, these results are in concordance with those from previous studies with *Epstein-Barr virus* [18], *Hepatitis C virus* [19], *Influenza A virus* H1N1 [20], and other viral and bacterial pathogens of mammals [8], [21]. These studies have shown that viral proteins preferentially target hub proteins in the human interactome. Herein, by assuming that virus targets are hubs, we investigate whether this information is propagated following the same scale of the plant interactome.

**Figure 1:**
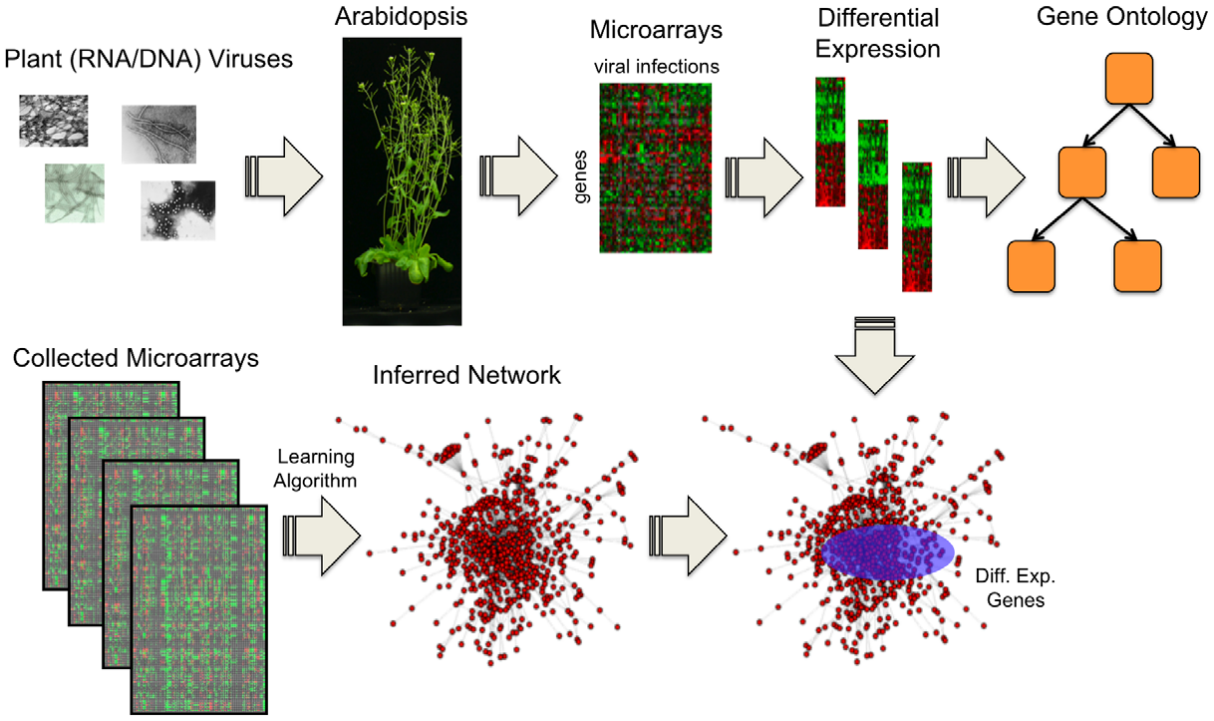
Overview of the Systems Biology approach we followed to study the viral infection in plants. We considered *A. thaliana* as model host. Microarray data from several infection experiments with viruses were collected to analyze the differentially expressed genes, and to perform functional analyses by harnessing GO annotations. In addition, by taking advantage of large databases of expression profiles derived from transcriptional perturbations, the global regulatory network of the host could be as a first approach unveiled by applying learning algorithms. The differential expression was then contextualized within the inferred network.

Microarray-based functional genomics, which provides a global view of transcriptional changes in host cells, has been the most commonly used method to study global changes during plant-virus interactions [4], [22]–[29]. However, the comparison of results obtained in distinct experiments involving different viruses is both complex and challenging; it has not been attempted in a systematic manner. Here, we present the results of a meta-analysis (Figure 1) of microarray data gathered from infections of the same host plant, *A. thaliana*, by seven plant RNA viruses belonging to four taxonomic families (*Tobacco etch potyvirus*, *Turnip mosaic potyvirus*, *Plum pox potyvirus*, *Tobacco mosaic tobamovirus*, *Tobacco rattle tobravirus*, *Turnip crinkle carmovirus*, and a laboratory-evolved strain of *Tobacco etch potyvirus*) and one DNA geminivirus (*Cabbage leaf curl geminivirus*) (Table 1). Using the same methodology, we first identified lists of genes that were up- and down-regulated, together with the sets of biological functions (gene ontology, GO) and metabolic pathways over-represented among them. These changes were then compared among the different virus infections, uncovering unexpected correlations within virus-specific phyla. In a second strategy, we explored these lists from a global network perspective, by mapping the altered genes onto different network models of the common host *A. thaliana*. This global computational approach unraveled a generic mode of interference by plant viruses, whereby perturbations incurred to the host interactome preferentially affect genes that are highly connected, central and form modules.

**Table 1:** List of viruses included in this study and some of their properties.

Virus	Taxonomy	Genome	Is *A. thaliana* a natural host?	
TEV	Family *Potyviridae*, Genus *Potyvirus*	Single stranded RNA, positive sense	No	
TEV-*At*17	Family *Potyviridae*, Genus *Potyvirus*	Single stranded RNA, positive sense	Yes (experimentally adapted to it)	
TuMV	Family *Potyviridae*, Genus *Potyvirus*	Single stranded RNA, positive sense	Yes	
PPV	Family *Potyviridae*, Genus *Potyvirus*	Single stranded RNA, positive sense	No	
TMV	Family *Virgaviridae*Genus *Tobamovirus*	Single stranded RNA positive sense	No	
TRV	Family *Virgaviridae* Genus *Tobravirus*	Single stranded RNA positive sense	No	
TCV	Family *Tombusviridae* Genus *Carmovirus*	Single stranded RNA positive sense	Yes	
CaLCuV	Family *Geminiviridae* Genus *Begomovirus*	Single stranded closed circular DNA	Yes	

## Results and Discussion

### Genetic Profile Targeted by Plant Viruses

Using transcriptomic data (steady-state RNA levels) extracted from 8 distinct virus infections on the model plant *A. thaliana*, we identified lists of genes with altered expression levels, referred herein to as ‘virus-responsive genes’ (or VRGs). These set of genes involves those genes that are directly or indirectly regulated by the virus and that are differentially expressed when the virus infects the cell. Those VRGs were then used to establish both general and specific genetic profiles associated to the pathogens of interest (File S1). We found that among the >22,000 genes inspected, a set of 5296 VRGs (2646 over- and 2650 under-expressed, respectively) is altered by at least one of the eight viruses studied. This VRG set may thus be used to reflect the global plant response to any viral infection. We found that the number of VRGs shared by more than one virus declines exponentially (Figure S1A and Figurer S1B). Seven VRGs were found up-regulated in common by six viruses, of which, surprisingly, six play a role in cell migration (*At3g57260*, *At5g10380*, *At3g14990*, *At3g28510*, *At5g52640*, and *At4g24690*) and one (*At1g75040*) encodes a PR-5 thaumatin-like protein, factors known for their involvement in pathogens responses. While no single VRG was identified in common among the eight infections, one VRG was systematically up-regulated by seven viruses (i.e., all except PPV) and found to encode an aspartyl protease involved, again, in cell migration in the diencepahlon (*At5g10760*). Three VRGs were down-regulated by six viruses, two of which correspond to different subunits of the NADPH dehydrogenase complex (*At1g18730* and *At5g58260*).

Not surprisingly, infections by the two different strains of TEV studied share the largest number of VRGs (197 over- and 282 under-expressed genes, respectively), although this may probably reflect, to some extent, homogeneity in experimental procedures. In the overlapping set, over-expressed genes principally have roles in response to stress (e.g., fungal resistance TIR-NB-LRR protein *At1g56510*, transcription factor *At1g22070*, U-box-domain-containing E3 ubiquitin ligase *At3g11840* that acts as a negative regulator of immune responses, or the aforementioned *At1g75040*), transport (e.g., the mitochondrial inner membrane translocase *At1g20350*, the high-affinity ammonium transporter *At2g38290*, or the glycolipid transfer protein *At4g39670*), transcription (e.g., the Myb-like transcription factor *At1g25550,* or the C2H2-type zinc finger *At3g46080*), and protein metabolism (e.g., the chaperone DnaJ-domain *At1g56300*, or the eukaryotic aspartyl protease *At5g10760*). The overlapping set of under-expressed genes is mostly composed of factors involved in basic metabolic and cellular processes (e.g., the member of the R2R3 factor *At1g18710*, the enzyme *At1g03630* that is NADPH- and light-dependent, or the α/β-hydrolase *At1g10740*).

Interestingly, a set of 27 VRGs was significantly over-expressed upon infections by the three viruses that naturally infect hosts from the *Brassicaceae* family (TuMV, TCV and CaLCuV) and by the TEV laboratory strain, which has been experimentally adapted to *A. thaliana* (TEV-*At*17); hereafter, we will refer to this set of four viruses as *Brassica*-infecting viruses. A common feature of these VRGs is that all of them play roles in stress response, including, among others, the disulfide isomerase *At1g21750* implicated in the regulation of apoptosis during endoplasmic reticulum stress as well as in osmotic stress. The set also includes the homolog of mammalian Bax inhibitor 1, *At5g47120,* which functions as an attenuator of biotic and abiotic stress-associated cell death, and the cytosolic heat shock protein *At5g52640*. The list further comprises several genes involved in signal transduction, such as the BAK1-interacting receptor-like kinase *At5g48380* that regulates multiple signaling routes for plant resistance, or the ATP binding kinase *At5g45800* involved in embryonic development. A set of 22 VRGs was also under-expressed in common, in plants infected by the *Brassica*-infecting viruses. This list includes, as in the afore-mentioned study of the two TEV strains, genes involved in central metabolic and cellular processes.

**Figure 2:**
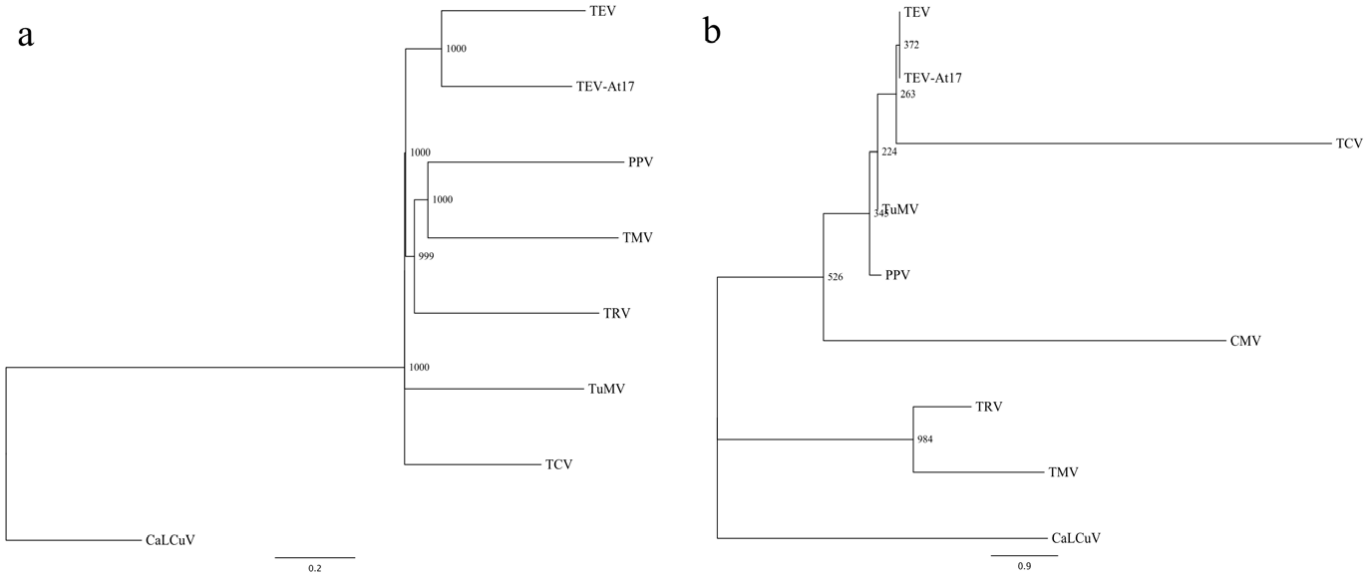
Phylogenetic relationships among viruses explain the similarities in gene expression. (A) Neighbor-joining dendrogram constructed using the similarity matrix computed from the lists of differentially expressed genes. Bootstrap support values are reported next to each node. (B) Maximum-likelihood phylogenetic tree constructed from the replicase genes of the seven RNA viruses included in the study. For CaLCuV, the Rep (replicase-associated protein) was used instead. The statistical quality of the different clusters was evaluated by bootstrap. Significance levels are shown next to each node.

Next, we sought to establish an overall comparison of the lists of VRGs identified from any of the eight viruses included in the analysis. To do so, we computed similarity scores among all pairs of lists, and constructed a dendrogram to visualize which viruses showed more closely related lists (Figure 2A). The eight viruses do not represent independent draws from a population; rather, some are phylogenetically related. It was therefore important to test whether the above overlap in VRGs reflected taxonomic correlations. In other words, do closely phylogenetically related viruses tend to share a higher number of VRGs, and does this overlap reduce as phylogenetic distance between viruses increases? To address this issue, we first used an alignment of the replicase genes from the eight viruses (the replicase-associated protein in the case of the geminivirus) to construct a maximum-likelihood phylogenetic tree (using the WAG + Γ model of amino acid susbstitutions and evaluating the significance of tree topology by 1000 bootstrap replicates; Figure 2B). Next we computed a congruency index [30] measuring the overlap between the tree topology obtained from the VRG similarity matrix (Figure 2A), on the one hand, and the topology of the estimated phylogenetic tree (Figure 2B), on the other. The congruence index (*I_cong_* = 1.4720) was significantly larger than expected by mere chance (*P* = 0.0052), suggesting that the two topologies are indeed highly congruent. This result supports the hypothesis that the overlap between VRG lists reflects the taxonomic relationships among viruses: two closely related viruses (e.g., the potyviruses TEV and TuMV) tend to alter the expression of a similar set of genes, whereas two non-related viruses (e.g., TEV and TRV) tend to alter different subsets of genes. The various viruses included in the study have distinct replication, gene expression, movement, and RNA silencing-suppression strategies that should somehow impact transcriptomic profiles differently. It is likely, however, that these strategies may be more conserved between phylogenetically related viruses than among viruses with weak or no phylogenetic relationship and, hence, the above study accounts for the differences and commonalities observed among virus phyla. This being said, convergent evolution in phylogenetically unrelated viruses may contribute to increase the overlap of VRG lists. For instance, potyviruses and carmoviruses employ overlapping RNA silencing-suppression strategies affecting the global metabolism of miRNAs, which may lead to a set of related host responses.

A potential weakness of the above meta-analyses of gene lists is that the different experiments not only differed in the methodological details and plant ecotypes, as described in the Materials and Methods section, but also in that different experiments took samples at different time points during the infection process and, in some cases, different tissues were also sampled. Ecotype-specific, time-dependent and tissue-specific responses to viral infection may turn on/off different subsets of genes [25], [31] and thus may not receive a high enough score to be classified as VRG according to the stringent statistical criteria used in the study. To minimize as much as possible these potential problems, only data from leaves were included in the present analyses, although the possible effect of ecotype and sampling time may still exist. We performed several statistical analyses to assess sources of errors in the data (see Materials and Methods), and we concluded that differences in ecotype or in sampling time would neither have a significant effect on the conclusions drawn from our meta-analyses.

### Integrative Functional Analysis

**Figure 3:**
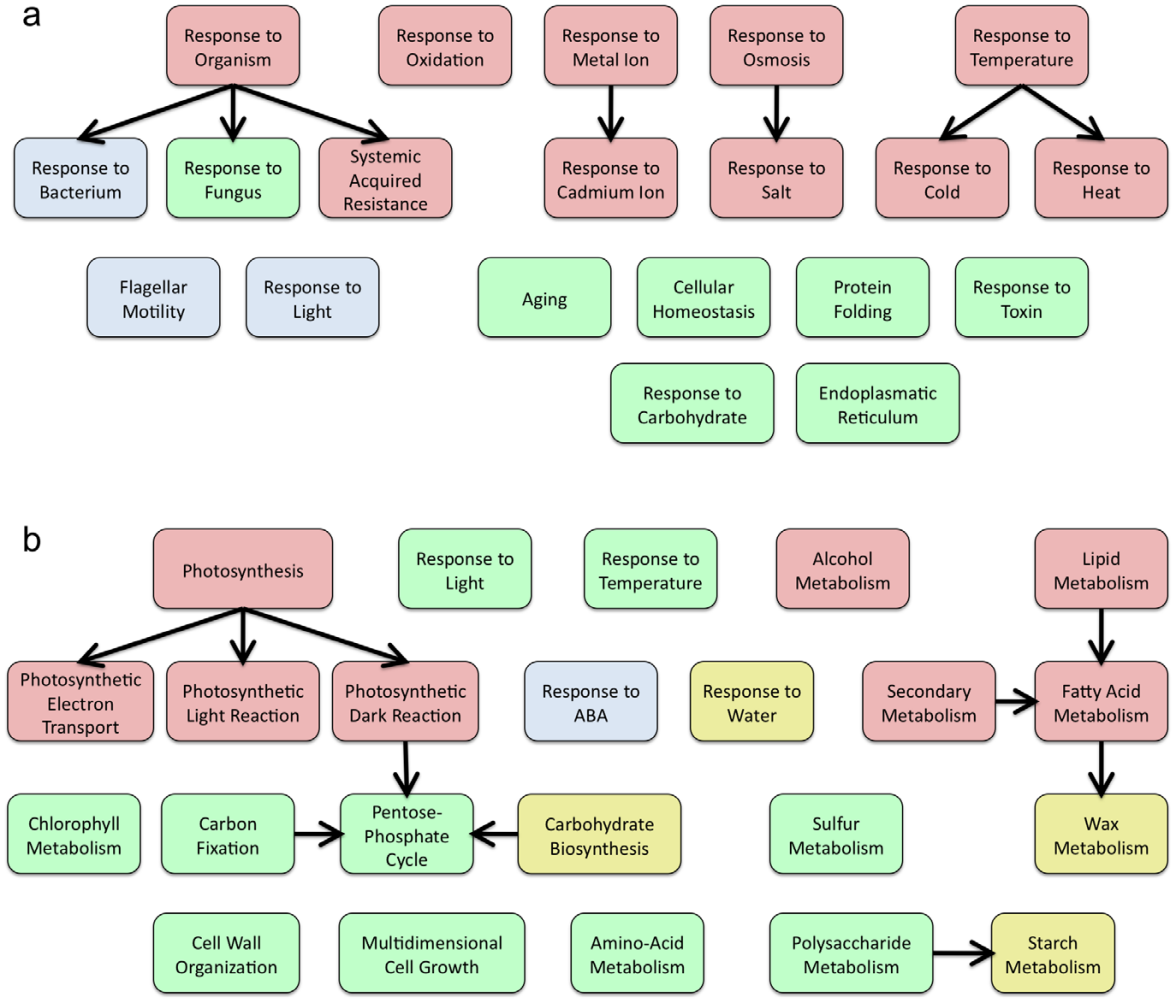
Functional analysis. (A) Over- and (B) under-expressed VRFs representing biological processes. In pallid red, VRFs present in at least five of the total eight viral infections (unspecific viral response); in pallid blue, VRFs in at least three of the four potyviral infections; in pallid green, VRFs in at least three of the four *Brassica*-infecting viral infections; in pallid yellow, common VRFs for *Potyvirus* and *Brassica*-infecting viruses.

Subsequently, we performed a functional analysis to map changes in gene expression onto regulations effecting global biological functions, thus establishing lists of ‘virus-responsive functions’ or VRFs (File S2). Figure S1C and Figure S1D illustrate the number of over- and under-expressed VRFs found in common in several viral infections. Two over-expressed VRFs are common to all eight viruses and encompass stress responses to temperature and to pathogens. By contrast, under-expressed VRFs, were mostly found to encompass different metabolic and photosynthetic processes (Figure 3 and Figure S2). In order to set aside changes in VRFs reflecting direct consequences of viral infection from those of indirect effects resulting, for instance, from cross-talks between biotic and abiotic stress, we partitioned data sets into (i) the unspecific response (i.e., VRFs significantly over-represented in at least five of the eight viral infections), (ii) the specific response to *Brassica*-infecting viruses (i.e., VRFs significantly over-represented in at least three of the four infections by *Brassica*-infecting viruses, eliminating those terms included in the unspecific response), and (iii) the specific response to *Potyvirus* (i.e., VRFs significantly over-represented in at least three of the four infections by potyviruses, eliminating those terms included in the unspecific response). This partitioned analysis notably confirmed that activation of systemic acquired resistance (SAR), an innate and salycilate-based immune response to pathogens, is indeed a general mechanism triggered by the exposure of plants to viruses (Figure 3). By contrast, down-regulation of polysaccharide metabolism, such as starch, appears as a specific response to *Brassica*-infecting viruses (Figure 3). This possibly explains why *A. thaliana* mutants compromised in starch biosynthesis display less severe virus-induced symptoms compared to wild type plants [32]. As it can be further observed in Figure 3, the specific response to *Brassica*-infecting viruses is larger than the unspecific one, suggesting that viruses impose additional constraints to their host through co-evolution to optimize their entire infectious cycle, by impelling the plant to introduce further gene-reprogramming sentences, notably within the immune response.

Figure S3 displays the neighbor-joining dendogram obtained from the similarity matrix computed from overlapping lists of significant VRFs obtained for the eight viruses. At the more stringent (i.e., 95%) bootstrap level, PPV, TMV and TRV form a distinctive cluster sharing several VRFs, which itself belongs to a significantly larger cluster that also includes the other three potyviruses (e.g., most of (+)ssRNA viruses) but excludes TCV. For those six viruses, over-expressed VRFs include responses to several abiotic stresses (e.g., temperature, osmotic, oxidative stresses) and defenses against pathogens, including SAR. Under-expressed VRFs in this large cluster include fatty acid metabolism and photosynthesis (Figure 3). CaLCuV is associated to a list of VRFs that differs significantly from that of the RNA viruses, as might be expected from the drastically distinct replication strategy of ssDNA viruses, which entails the reactivation of the host DNA replication machinery. Interestingly, TCV occupies an intermediate position in the dendrogram, close to the base of the cluster formed by the other (+)ssRNA viruses.

As in the previous section, we tested if the dendrogram topology shown in Figure S3 was congruent with the phylogenetic history of the viruses (Figure 2B). In this case, the congruence index (*I_cong_* = 1.2267) did not significantly differ from what was expected by chance (*P* = 0.1005), thus suggesting that both topologies are not highly congruent; in other words, that the set of VRFs altered by two related viruses is similar to the one altered by two non-related viruses. At first, this result may be seen as contradicting the previous one, obtained by comparing lists of VRGs. Broad GO terms, however, encompass a multitude of genes and it may well be that different viruses affect the same VRF by modifying the expression of different target genes. Consistent with this idea, TEV and TRV infections were both associated with a significant over-representation of the GO term ‘stress response’. In both cases, the number of VRGs connected to this specific GO is similar (108 for TEV and 93 for TRV); yet only four genes are affected in common between the two viruses. Therefore, this analysis reveals that comparable trends in the global reprogramming of cellular functions might be achieved via highly dissimilar gene expression changes induced by distinct viruses.

### Metabolic Pathways Targeted by Viruses

Next, a metabolic pathway analysis was conducted to uncover the biochemical networks that were over- and under-expressed by viral infections. To that aim, we represented the global metabolic map of *A. thaliana* infected with a given virus by overlaying the expression of the corresponding enzymes. To contextualize the information of all viruses in a single picture, we constructed, on the one hand, a list of genes that characterize the unspecific virus-plant interaction (VRGs in at least five of the eight viral infections studied), and, on the other hand, a list of genes that depict the interaction between *Brassica*-infecting viruses and *A. thaliana* (VRGs in at least three of the four infections by *Brassica*-infecting viruses, eliminating those genes included in the unspecific response). The results presented in the Figure S4 show that the metabolic pathways over-represented as part of the unspecific response to viruses notably included cellulose biosynthesis -required for cell wall integrity- and nitrogen fixation. In addition, *Brassica*-infecting viruses were found to distinctively induce the biosynthesis of cytokines, a family of hormones central to plant development and growth, also involved in detoxification [33]. *Brassica*-infecting viruses also specifically induced the Rubisco shunt, which is a more efficient converter of carbohydrates into acetyl-CoA than glycolysis [34]. The unspecific gene down-regulation response as a part of the viral reprogramming of central metabolism was found to affect the Calvin cycle and glycolysis. This observation is consistent with the previously proposed idea [35] that viruses impel the plant to redirect resources towards immune systems and, in particular, biotic stress responses, to the detriment of developmental processes. As a result, increased tolerance to viral infections might be achieved. Biosynthesis of starch -the major energy reservoir in the cell- photorespiration, and fatty acid biosynthesis are some of the biochemical routes that were found to be specifically down-regulated by *Brassica*-infecting viruses. In addition, *Brassica*-infecting viruses induce symptoms such as chlorosis and spotting, leaf curling and overall dwarfism, whereas, apart from a minor delay in growth, the other viruses progress asymptomatically. All together suggests that viruses more adapted to their hosts impose a more stringent rerouting of the plant metabolism, which may result in more severe symptoms and/or increased viral virulence.

### Viruses Preferentially Alter Highly Connected Central Genes

Next we focused on the impact of viruses on two different predicted global networks of *A. thaliana*: a transcriptional regulatory network (TRN) [36] and a protein-protein interaction network (PPIN) [37]. A second TRN (TRN2; see Material and Methods) and the graphical Gaussian model of interaction network (GGIN) [38] (File S3) were also added to consolidate this study. Very recently, a new collection of about 11,300 experimentally predicted PPIs has been released [39], although this interactome is too small to perform our analyses in a meaningful manner. The PPIN here considered accounts for almost the 30% of such PPIs, and for future studies the two interactomes will be combined. Both TRN and PPIN have the properties of scale-free and small-worlds, the two major characteristic properties of real biological networks [13], [15], [16]. A network is named scale-free if a node selected randomly has a number of links (connectivity) that follows a specific mathematical function refereed to as the power law, implying that the network has no characteristic scale and is self-similar [13], [40]. A network has the property of a small-world if any two nodes are connected by a small number of edges; mathematically, this number should grow as a power of the number of nodes in the network [15], [41].

**Figure 4:**
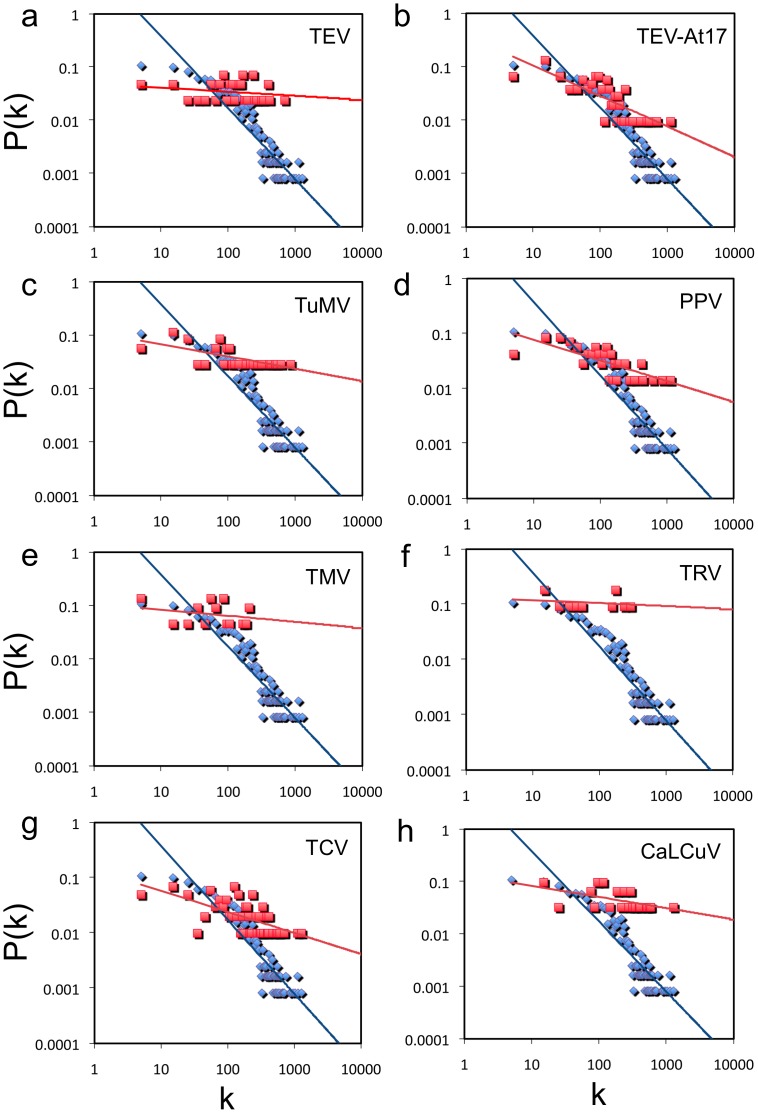
Outgoing connectivity distributions. Distributions are contextualized in the TRN, for the VRGs (red) and the whole interactome (blue).

**Figure 5:**
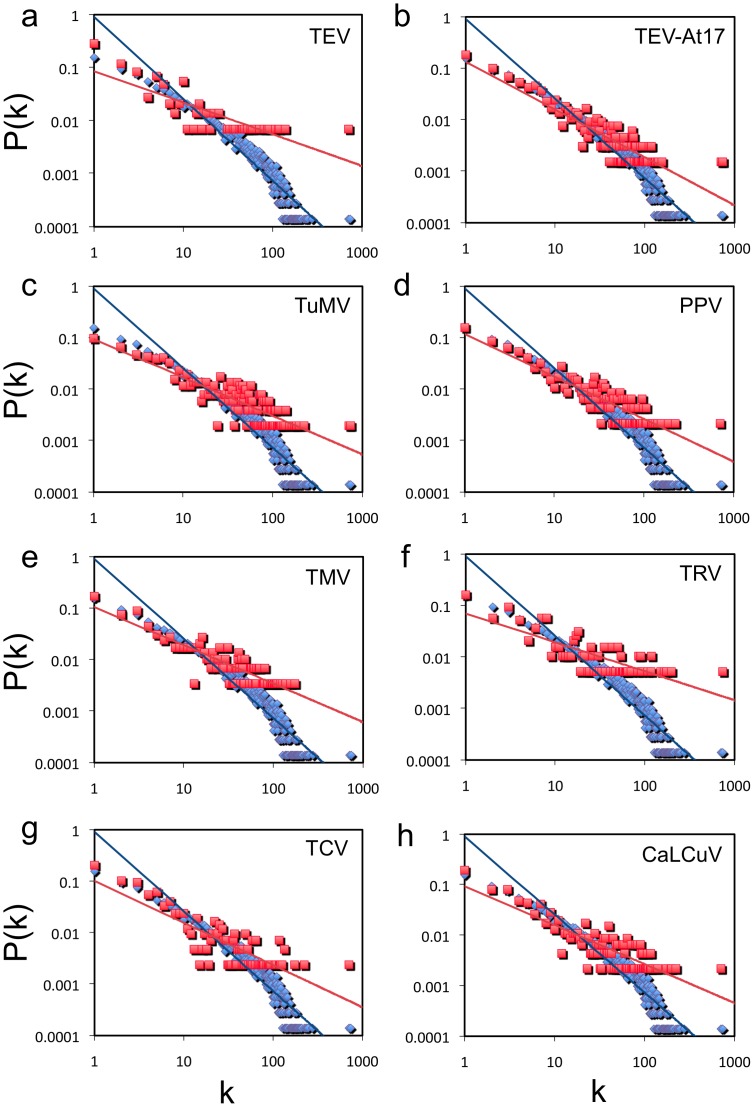
Connectivity distributions. Distributions are contextualized in the PPIN, for the VRGs (red) and the whole interactome (blue).

First, we analyzed the connectivity distribution for the VRGs as compared to the global set of genes. Roughly, if those genes were located in the periphery of the network, their connectivity would be expected to be smaller than if they were central, since the network is scale-free (Figure S5). As the TRN is a directed network, we focused on the outgoing connectivity, that is, the number of regulations of a given transcription factor with its targets in the network. In Figure 4 we show the connectivity distributions for all viral infections. Table 2 summarizes the value of the power-law exponent that better fits this particular distribution as well as the average connectivity. Of course, the degree distributions are not perfect power-laws and could be better explained by truncated power-laws or Weibull distributions [42], [43] or even by a mixture of several Poisson distributions [44]. However, here we are not concerned to propose a precise model for the distributions. For our purpose, the computation of the slope fitting to a power-law or the computation of other parameters derived from more complex distributions would not change the conclusions. To statistically assess differences between the VRGs and the global set of genes, we used *t*-tests for differences in slopes and Mann-Whitney tests for differences in the location of the high-degree genes within the distributions. We also performed for all cases a Kolmogorov-Smirnov test to assess the difference of the distributions, and then we combined the resulting *P*-values in an overall test of goodness of fit (Fisher’s method, χ^2^ = 61.5 for the TRN and χ^2^ = 101 for the PPIN; in both cases, 16 d.f., *P*<0.0001). No significant differences were found between the incoming connectivity distributions of VRGs and the one characterizing the whole network (Figure S6). Figure 5 shows the corresponding connectivity distributions using the PPIN. We found that, in all cases, i.e. whether considering the TRN or the PPIN, the slope of the fitted power-law distributions of VRGs was significantly smaller than the slope calculated for the fitted distribution of the whole interactome. These results were also confirmed by analyzing the TRN2 (Figure S7) but not upon analysis of the GGIN (Figure S8). In this vein, the Mann-Whitney tests showed, for almost all viruses in both TRN and PPIN, that the mean of the high-degree VRGs is significantly greater than the mean of the total high-degree genes (Table 2), which is in tune with the observation of a smaller slope in the degree distribution of VRGs. Apart of that *Brassica*-infecting viruses affect on average more genes, these viruses also manipulate more interactions, irrespective to the network model (one-tailed *t*-test, *P*<0.05). However, we did not find any significant difference among viruses in terms of the average connectivities and power-law distribution exponents. We conclude that a smaller slope of the power-law distribution is a general trend characterizing the VRGs, indicating that viral infection preferentially alters the expression of highly connected genes (hubs) rather than random genes within the whole network. This could reflect a cellular response to gain robustness against the manipulation of the host by viruses.

**Table 2:** Summary of topological properties of the differentially expressed VRGs from several viral infections contextualized in the *A. thaliana* TRN and PPIN.

	VRGs		VRFs		TRN			PPIN			
	Over	Under	Over	Under	Edges	γ(*P* ^a^)	〈*k*〉 (*P* ^b^)	Edges	γ (*P* ^a^)	〈*k*〉 (*P* ^b^)	〈*b*〉×10^−4^ (*P* ^b^)
**TEV**	356	322	35	41	1275	0.07 (<10^−4^)	162 (0.02)	64	0.59 (<10^−4^)	18 (0.18)	9.56 (10^−4^)
**TEV-** ***At*** **17**	950	1441	32	90	2850	0.57 (<10^−4^)	115 (0.23)	881	0.92 (<10^−4^)	22 (0.03)	5.72 (<10^−4^)
**TuMV**	754	390	29	30	1034	0.23 (<10^−4^)	172 (2·10^−4^)	1665	0.74 (<10^−4^)	34 (<10^−4^)	8.63 (<10^−4^)
**PPV**	747	740	98	8	945	0.37 (<10^−4^)	153 (5·10^−3^)	535	0.82 (<10^−4^)	24 (0.02)	6.46 (<10^−4^)
**TMV**	498	225	62	0	67	0.11 (<10^−4^)	76 (1)	214	0.74 (<10^−4^)	22 (0.15)	3.35 (0.40)
**TRV**	215	284	14	26	82	0.05 (<10^−4^)	111 (0.45)	154	0.56 (<10^−4^)	26 (5·10^−4^)	8.20 (<10^−4^)
**TCV**	708	846	91	70	4328	0.38 (<10^−4^)	188 (<10^−4^)	364	0.81 (<10^−4^)	19 (0.04)	5.50 (3·10^−4^)
**CaLCuV**	454	732	66	107	2117	0.21 (<10^−4^)	255 (<10^−4^)	664	0.77 (<10^−4^)	24 (<10^−4^)	6.14 (<10^−4^)
**Interactome**	–	–	–	–	139,440	1.33 (−)	114 (−)	72,266	1.54 (−)	20 (–)	3.43 (−)

We show the number of VRGs and VRFs (over- and under-expressed), the number of interactions (edges) manipulated by the virus, the power-law distribution exponent (for connectivity γ), the average connectivity (*(k)*), and the average betweenness (*(b)*). We also show the P-value for the tests comparing the shape and location of the VRGs distributions with respect to the corresponding whole interactome (aStudent *t*-test, bMann-Whitney *U*-test).

A more global scale analysis involved calculation of the betweenness centrality distribution, understood as the relative number of shortest paths traversing a given gene. Table 2 summarizes the values of the average betweenness for these sets of VRGs. The betweenness values were relative in all cases to the global network. In addition, for each subnetwork generated with the VRGs, we computed the total number of shortest paths present in it, the distribution of path lengths, and their characteristic length (Figure S9, Figure S10 and Figure S11, respectively). Since PPIN represents the case of an undirected graph, we restricted the analysis to this interactome to evaluate the betweenness of the VRGs. For each gene we have a value of betweenness, so we can compute the average value for the VRGs and then compare it with the one obtained for the total set of genes. We found that, as occurred with the connectivity at the local level, the VRGs were significantly central for seven out of eight viruses, with average betweenness centrality values significantly greater than observed for the average of the whole interactome (Figure S12A; Mann-Whitney test *P*<0.05). TMV was the exception. We also found that betweenness and connectivity are significantly positively correlated (Figure S12b; Spearman *ρ* = 0.8885, 10,286 d.f., *P*<0.0001, releasing the isolated nodes), despite the high variability of betweenness at low connectivity values, a characteristic of hierarchical networks.

With our study, we do not explicitly prove a physical interaction between hubs and viral proteins, but we demonstrate that the hubs of the plant interactome are over/under-expressed after viral infection. These hubs mediate the immune response to produce large changes in the genetic profile, but they are redundant in this process. Furthermore, Uetz *et al.* [45] found that, whereas the PPINs of *Herpesviruses* lack scale-free and small-world properties, the topology of such viral networks completely change from a highly coupled module to a more typical scale-free network of interacting submodules when integrating the interactions with human proteins. Available data from yeast two-hybrid experiments [46]–[50] allowed us to further infer a PPIN for *Potyviruses* (Figure S13). This inferred PPIN shows that all 11 potyviral-encoded proteins are highly connected and that, similar to the herpesviruses case, the underlying network is not scale-free.

### Modular Organization of Virus-altered Genes

**Figure 6:**
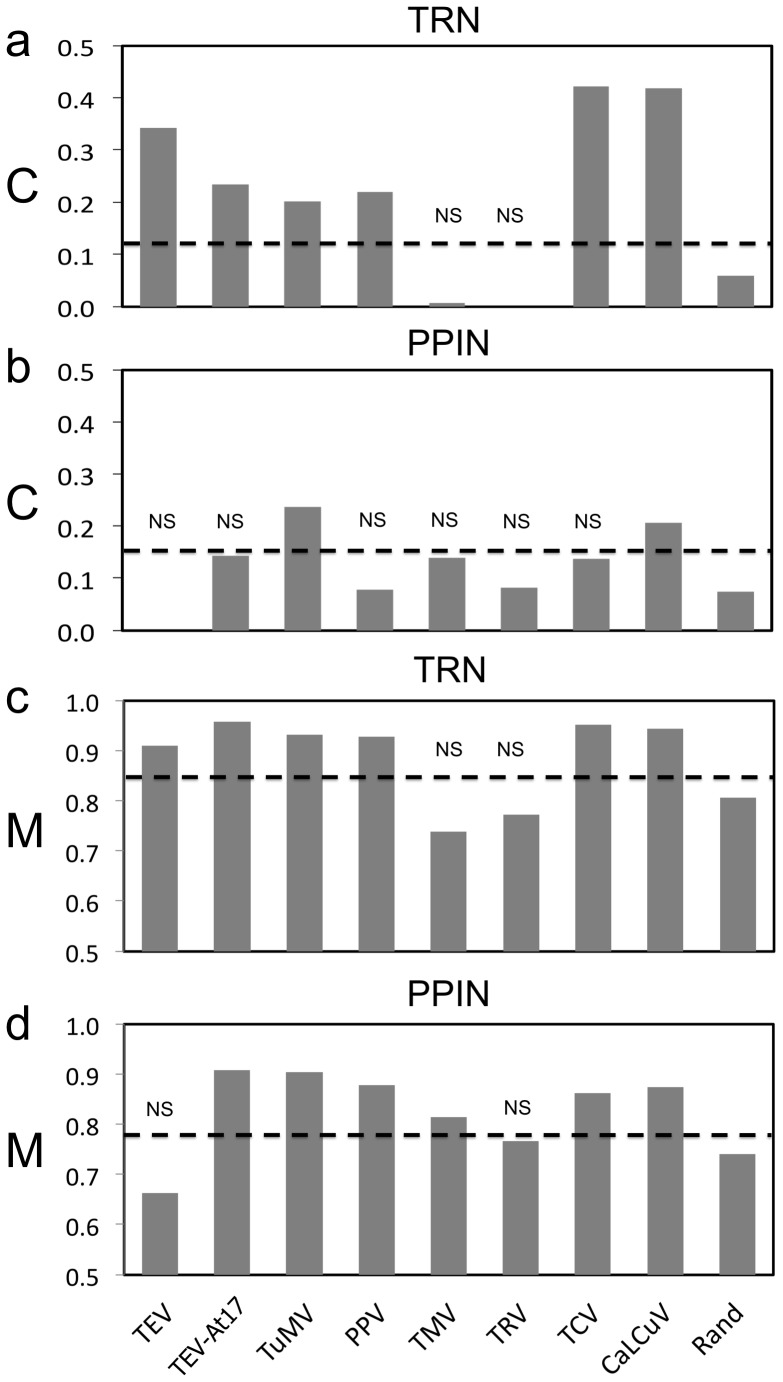
Measures of subnetwork organization. (A, B) Clustering (*C*) and (C, D) modularity (*M*) coefficients for the subnetworks generated by the VRGs, contextualized in the TRN and PPIN. *Rand* indicates the average value for random subnetworks (100 replicates). NS denotes non-significant value following a one-tailed *z*-test. Horizontal dashed lines represent the cutoff value for statistical significance.

We further analyzed the subnetworks generated from the VRGs according to the different global networks by focusing on two important topological properties: clustering and modularity. Noteworthy, the relatively smaller lists of genes available for TMV and TRV infections (owing to the type of microarray used) are to be treated with caution, as the corresponding analyses for the two viruses may reflect non-significant results. In Figure 6A and Figure 6B, we show the clustering coefficient for all subnetworks. According to the TRN, almost all subnetworks are tightly clustered (also validated for the TRN2 and GGIN, Figure S14). However, only the subnetworks for two viruses (TuMV and CaLCuV) are clustered according to the PPIN, suggesting that a disruption of the transcriptional organization could be more advantageous for the two viruses. To further support this clustering analysis, we computed the assortativity coefficients that characterize those subnetworks, in this case only focusing on transcription factors (Table S1). Assortativity refers to a preference for a network’s node to attach to others that are similar (assortative) or different (dissortative) in some way. We found that TRN-based subnetworks are essentially assortative, whereas PPIN-based ones are mostly dissortative (a likely consequence of the scale-free topology). Thus, regulations between transcription factor-related VRGs behave like in metabolic or social networks, in which hubs tend to be connected among them [40], [51]. We further studied the modularity properties of those subnetworks, understood as a decomposition metric based on the number of connected components present in the subnetwork (Figure S15), which is a less stringent clustering parameter. Remarkably, we found that in both TRN and PPIN, VRGs display a modular arrangement (Figure 6C and Figure 6D) for all infections except with TRV and TMV (a possible reflection of the much more modest number of genes analyzed) and the same result was obtained using the TRN2 and GGIN (Figure S16). We conclude that, in general, the virus tends to induce the differential expression of genes that are clustered (linked among them on the resulting subnetwork) and belong to local modules, rather than randomly interacting with genes sparsely distributed in the network.

## Conclusions

The results of our meta-analyses combining transcriptomic data gathered for eight different viruses all infecting a common host, *A. thaliana*, confirm that host cells undergo significant reprogramming of their transcriptome during infection, which is likely a central requirement for turning on host defenses. Rather than focusing on the details of each virus infection, however, our study was designed to uncover generic features defining either the host response to, or the targets manipulated by, the various viruses tested. We found that the overlap in the lists of genes whose expression is altered upon infection (VRGs) decreases as the phylogenetic distance between the viruses increases, thus suggesting that related viruses may interact with similar host components, whereas non-related viruses may manipulate different targets. This association at the VRG level does not hold, however, at the level of altered, global biological functions (VRF), thus suggesting that a common set of overall functional responses to infection may result from the manipulation of sometimes drastically different target genes.

One caveat of the meta-analysis studies such as the one reported here, however, is that they are conservative in design. They identify responses that are strong enough to be detected against the intrinsically high noise level as a consequence of the diversity of viral systems and microarray platforms used in the original studies that served as the basis for the present one. While reductionism through single-cell transcriptome analyses has been successfully employed in virus-infected mammalian cell cultures [9] and in plant protoplasts [29], studying *in vivo* virus-host interactions obviously adds many layers of complexity and variability, which are clearly reflected here. Nonetheless, our study shows that such complexity does not, *a priori*, constitute an insurmountable obstruction to the discovery of generic patterns associated to plant viral infections. In addition, our methodology was based on standard techniques to capture differential gene expression. The use of experimental protocols accounting for many replicates (both biological and technical) helps minimizing errors in the identification of VRGs. Once these genes are identified, the GO analysis could provide a functional picture, although it is true that small sets of genes tend to produce non-significant results. Moreover, the construction of the host network models also has the associated error to statistical inference. Since networks are inferred from experimental data, or even in combination with alignments of sequences and further computational techniques, a tradeoff between coverage and precision must be achieved. The selection of optimal networks then introduces a given number of false positives that may divert the topological properties of VRGs (connectivity, betweenness, clustering, assortativity, and modularity). Efforts for constructing large databases of reliable interactions would enhance the predictability of such computational studies. Because the uncertainty introduced in the network is multiplicative to the errors that come from the identification of VRGs, the results derived from this computational pipeline are not entirely free of false positives. Nevertheless, we expect our conclusions to be robust to these accumulated errors due to the delicate treatment of the raw data prior to any further analyses, the large number of statistical tests performed to ensure homogeneity in data across experiments, and the consistency of the results to changes in assumptions (e.g., using different models of networks).

Our study points out that VRGs are, in general, more highly connected, central and modular than expected by chance. This result agrees with the fact that viral proteins preferentially interact with hub regulator genes [17]–[21], [45], although VRGs not necessarily entail virus targets. Probably as a plant strategy, through hub genes the signal can be disseminated at large to change the whole genetic profile. Then, even a small number of viral proteins can affect a considerable number of host genes. In the case of *Potyviruses*, 11 mature proteins provoke significant changes in expression in about a thousand of host genes. That more hub genes (both from TRN and PPIN) than expected by chance were differentially expressed indeed reflects an effect of the virus over them, and also indicates that the information flow from virus targets to immune response proteins is strengthened *ab initio* (lower slope in the power-law degree distribution). We therefore hypothesize that this over-triggering of hubs is a mechanism that confers robustness to the plant to express the immune system. Whether a virus deactivated a recognition pathway, redundant hubs would emerge to counteract this viral action. We have confirmed this observation for all the plant viruses included in our meta-analysis, thus uncovering a possible universal pattern in virus-host interactions. A second general pattern emerging from animal virus studies is that the topological properties of viral infections differ when considering only viral proteins (e.g., using yeast-two hybrids experiments) than when they are considered in the context of PPIN from the host cell [45]. Here, we have not been able to test this property owing to the lack of adequate information. A future challenge in plant virology research will be to combine data sets from yeast two-hybrids or BiFC studies, transcriptomic experiments and carefully curated literature surveys, in order to reveal the specific interactions between plant and virus proteins and the effect of such interactions on viral PPIN topology. *In vitro*-reconstructed interactomes [17], [39] by themselves do not capture all the biological features of the viral infection, so *in vivo* data, while adding more complexity, are essential for further studies.

## Materials and Methods

### Plant Viruses

In this work, we studied the mode of action of seven positive-sense single-stranded RNA viruses (Baltimore’s group IV) and of one virus whose genome is composed by a single-stranded circular ambisense DNA molecule (Baltimore’s group II) on a common plant host, *A. thaliana* (Table 1). The set of RNA viruses is formed by *Tobacco etch virus* (TEV), *Turnip mosaic potyvirus* (TuMV), *Plum pox potyvirus* (PPV), *Tobacco mosaic tobamovirus* (TMV), one *Tobacco rattle tobravirus* (TRV), and *Turnip crinkle carmovirus* (TCV). In addition, we considered a laboratory-evolved strain of TEV (TEV-*At*17), which was obtained after 17 serial passages in *A. thaliana* [27]. TEV-*At*17 shows higher fitness and produces more severe symptoms in *A. thaliana* than the ancestral TEV strain. The ssDNA virus included in the study was *Cabbage leaf curl begomovirus* (CaLCuV).

### Transcriptomic Data

TEV and TEV-*At*17 expression data (two-color raw data, five replicates for each, NCBI GEO accession GSE11088) were obtained from ecotype L*er*-0 plants 14 days post-inoculation (dpi) [26], [27]. TuMV data (Affymetrix raw data, three replicates, ArrayExpress accession e-mexp-509) were obtained 5 dpi from ecotype Col-0 plants [25]. These three data sets were normalized using the RMA method [52] for background correction and quantiles for array scaling, and the list of differentially expressed genes was obtained by performing a Limma test [53] with a correction for multiple testing using the false discovery rate (FDR) procedure [54] (adjusted *P*<0.05). PPV data (Affymetrix preprocessed data, three replicates, NCBI GEO accession GSE11217) were obtained 17 dpi from Col-0 plants [29]. In this case, data normalization was done using the Affymetrix MAS 5.0 software package, and the differential expression using a one-way ANOVA test with a correction for multiple testing using the FDR procedure (adjusted *P*<0.05), followed by a fold-change criterion of 1.5 in *z*-score over all genes (averaging replicates). TMV data (two-color raw data, five replicates, deposited in www.bio.puc.cl/labs/arce/index.html) were obtained from ecotype Uk-4 plants 10 dpi [24], and normalized using the RMA method for background correction and quantiles for array scaling. The list of differentially expressed genes was obtained by performing a fold-change criterion of 1.96 in *z*-score over all genes (averaging replicates). TRV data (two-color raw data, three replicates (dye-swap), NCBI GEO accession GSE15557) were measured 8 dpi from Col-0 leaves. TCV data (two-color raw data, three replicates, NCBI GEO accession GSE29387) were quantified 10 dpi in Col-0 plants. These two data sets were normalized using the CATMA BGS procedure [55], and the list of differentially expressed genes was obtained by performing a Limma test with FDR correction (adjusted *P*<0.05). In addition, for TCV data, a fold-change criterion of 1.96 in *z*-score over all genes (averaging replicates) was applied. Finally, CaLCuV data (Affymetrix raw data, three replicates, ArrayExpress accession E-ATMX-34) were collected from Col-0 plants 12 dpi [28]. These data were normalized using subtraction for background correction and LOWESS [56] for array scaling, and the list of differentially expressed genes was obtained by performing a mixed ANOVA test with a correction for multiple testing using the FDR procedure (adjusted *P*<0.05). To perform the data normalization and to obtain the differentially expressed genes, we used the GEPAS tool [57], which is implemented within the BABELOMICS webserver [58]. We would like to notice at this point that the variability in the normalization methods comes from the lack of source data files that prevents incorporating them into a common platform, although we have intended to provide a homogeneous compendium as much as possible.

### Validity of Meta-analysis

The heterogeneity in the host ecotype used in different experiments (L*er*-0 for TEV and TEV-*At*17, Uk-4 for TMV and Col-0 for the rest) and in the time at which samples were obtained (ranging from 5 to 17 dpi) may weaken the conclusions from a meta-analysis. Therefore, to evaluate the robustness of our results, we first tested for the effect of these two variables. First, for each gene that showed a significant alteration in its expression level in at least one of the eight viral infections (a total of 6546 genes), we sought whether the observed differences grouped according to the plant ecotype used in the experiments. For this, we classified experiments into two categories: those performed in Col-0 *versus* those not performed in Col-0. Only seven genes (*At1g14970*, *At1g50250*, *At1g78170*, *At2g16700*, *At2g20780*, *At3g45860*, and *At4g12520*) had expression levels that were significantly affected by the host ecotype (Mann-Whitney test). However, if a correction for multiple testing (FDR procedure; adjusted *P*<0.05) was used, none of these seven genes remained significant. Second, for each of the 6546 altered genes, we sought whether expression levels classified according to sampling time. Sampling times were ranked into three categories (early, between 5–8 dpi; intermediate, between 10–12 dpi; and late, in the range 14–17 dpi). In this case, 54 genes showed a significant effect of the sampling time (Kruskal-Wallis test), although none of them remained significant after applying the more stringent FDR procedure. In addition to this test, we also sought for significant correlations between expression levels and sampling time (Pearson correlation coefficient). In this case, 96 genes showed a significant correlation (either positive or negative), although none of them remained so after the FDR correction. Therefore, we conclude that differences in ecotype or in sampling time would neither have a significant effect on the conclusions drawn from our meta-analyses. Nonetheless, the conclusions from our meta-analyses should be taken conservatively.

### Functional Analysis

For each list of “virus-responsive genes”, or simply VRGs, (over- or under-expressed), we looked for the significant over-represented biological processes (GO terms between levels 3 and 9, referred in the text as VRFs – or virus-responsive functions–) within that list. The statistical significance was evaluated by means of a Fisher's exact test for 2×2 contingency tables with a correction for multiple testing using the FDR procedure (adjusted *P*<0.05). To perform the functional analysis of the VRGs, we used the FatiGO tool [59], implemented in the BABELOMICS webserver. At the metabolic level, we overlaid the expression data for each viral infection into the global metabolic map of *A. thaliana*. To visualize the map we used the TAIR AraCyc tool [60].

To quantitatively evaluate the similarity in the lists of VRGs and of the corresponding biological functions, we performed a hierarchical clustering analysis by constructing neighbor-joining dendrograms using the program NEIGHBOR from the PHYLIP v3.6 package (http://evolution.genetics.washington.edu/phylip.html). The similarity matrix ***S*** was defined by using the metric *S_ij_* = 2*N_ij_*/(*N_i_* + *N_j_*), where *N_i_* and *N_j_* are the total number of genes (or GO terms) whose expression is altered upon infection with virus *i* and *j*, and *N_ij_* the number of genes (or GO terms) altered by both viruses *i* and *j*. Statistical significance of the different clusters was evaluated by bootstrapping the gene (or GO) lists (based on 1000 pseudoreplicates).

### Genomic Interaction Networks

The transcriptional regulatory network (TRN) was previously published by Carrera *et al.* [36]. In short, the model was inferred using a reverse-engineering procedure, based on mutual information with a local significance (*z*-score computation) as estimator of the likelihood, for capturing coexpression patterns between transcription factors (TFs) and genes, and has optimal levels of confidence and coverage. This network contains 139,440 TF-gene interactions and involves 19,108 genes. For the protein-protein interaction network (PPIN), we used the release 2.0 of the *A. thaliana* predicted interactome available for downloading at TAIR (www.arabidopsis.org). This network consists of a set of 72,266 predicted interactions involving 7177 proteins, of which about 3000 interactions are experimentally confirmed (merging datasets from TAIR, IntAct-EBI, and BIND/BOND). In short, the prediction algorithm [37] began with the identification of orthologs of *A. thaliana* proteins in seven other species (*Escherichia coli*, *Saccharomyces pombe*, *Saccharomyces cerevisiae*, *Caenorhabditis elegans*, *Drosophila melanogaster*, *Mus musculus*, and *Homo sapiens*) for which partial interactions existed. Next, an interaction was predicted to exist in *A. thaliana* if it was described for any of the seven species. Notice that TRN is directed, whereas PPIN is undirected. In addition, we considered a smaller transcriptional network (TRN2), with high confidence and low coverage, that contains 18,446 TF-gene interactions and links 7108 genes [36], and the graphical Gaussian interaction network (GGIN) model previously published by Ma *et al*. [38] that contains 21,101 effective gene-to-gene interactions involving 6722 genes, where coexpression patterns between gene pairs were evaluated according to a conditional correlation.

### Topological Analysis

To analyze the impact of a viral infection in terms of genetic interactions, we studied the principal topological properties on the inferred networks: connectivity, clustering, connected components, shortest paths and modularity. For each VRG (up and down), we collected its connectivity and betweenness centrality, according to the global interactome. Differences in connectivity (*k*) and betweenness (*b*) among the VRGs and the total set of plant genes were analyzed by means of one-tailed Mann-Whitney *U*-tests (*P*<0.05) considering the superior tails of the distributions (i.e., the genes satisfying *k* > 〈*k*〉 or *b* > 〈*b*〉) [19], [61]. Furthermore, we performed linear regressions in the log-log space to obtain the critical exponents, *γ*, of the power-law degree distribution *P*(*k*) *∼ k^−γ^* and assessed the statistical significance of the inferred values using Student *t*-tests (*P*<0.0001). In addition, for each virus we generated the corresponding subnetwork by selecting those VRGs, releasing those isolated nodes. Random subnetworks were also constructed to perform statistical significance tests. The clustering coefficient (*C*), the assortativity coefficient (*A*), the number of connected components (*CC*), and the shortest path (*SP*) distribution were computed to characterize the subnetworks. Here, *A* was defined as the slope of the linear regression between the connectivity of a node and the average connectivity of its neighbors, being a network assortative when *A* >0. Moreover, we defined a modularity coefficient (*M*), less stringent than the clustering one, given by , (*sensu* Shannon entropy in information theory) where *N_c_* is the number of genes in the connected component *c*, and *N* the total number of genes in the subnetwork [62]. Accordingly, *M* = 1 in case of just one *CC*, whereas *M* tends to 0 as the number of *CC* increases. To assess the statistical significance of the results, we performed a one-tailed *z*-test with a confidence level of 95% (*z* >1.64) over 100 random subnetworks.

## Supporting Information

Figure S1 **Altered gene expressions and GO terms.** Distribution of genes up/down-expressed (A and B) and GO terms over/under represented (C and D) in *A. thaliana* after infection with the number of viruses indicated in the ordinates axis. The distributions are the result of comparing the differential patterns *a posteriori* between several viruses.
(TIFF)

Figure S2 **Altered VRFs.** Summary of (red) over- and (blue) under-expressed VRFs representing biological processes. In black, consensus of VRFs for any viral infection (unspecific viral response). In white, consensus of VRFs specifically altered by *Brassica*-infecting viruses.
(TIF)

Figure S3 **Neighbor-joining dendrogram constructed using the similarity matrix computed using the lists of over-represented GO terms.**
(TIF)

Figure S4 **Metabolic map of** ***A. thaliana.*** Highlighted the reactions altered by the unspecific viral response (VRGs in at least five of the total eight viral infections) and the specific *Brassica*-infecting virus response (VRGs in at least three of the four infections by *Brassica*-infecting viruses). Red (unspecific) and yellow (specific to *Brassica*-infecting viruses) reactions are over-expressed, whereas blue (unspecific) and green (*Brassica*-infecting) reactions are under-expressed. BS means biosynthesis, and TR, transformations.
(TIFF)

Figure S5 **Predicted connectivity distribution for the whole plant interactome (red line) and the subnetwork generated by the total differentially (up/down) expressed VRGs (blue line).** The set of VRGs can be in the periphery of the interactome if they have low connectivity (a) or in the core in they are highly connected (b).
(TIFF)

Figure S6 **Incoming connectivity distribution.** The distribution is contextualized in the TRN interactome, for the VRGs (red), and the whole interactome (blue).
(TIFF)

Figure S7 **Outgoing connectivity distribution.** The distribution is contextualized in the TRN2 interactome, for the VRGs (red), and the whole interactome (blue).
(TIFF)

Figure S8 **Connectivity distribution.** The distribution is contextualized in the GGIN interactome, for the VRGs (red), and the whole interactome (blue).
(TIFF)

Figure S9 **Statistics for the shortest paths.** Shortest path average (A, C, E, and G) and total number of shortest paths (B, D, F, and H) for the subnetworks generated by the differentially expressed genes from several viral infections contextualized in different interactomes. *Rand* indicates the average value of randomly selected gene lists. Inter stands for interactome. For the TRN and TRN2 interactomes, we considered undirected edges; otherwise, the number of shortest paths is very low.
(TIFF)

Figure S10 **Shortest path distribution.** Contextualized in the TRN interactome, for the subnetwork generated by the differentially expressed genes after viral infection (A, B, C, D, E, F, and G), and by lists of randomly selected genes (H). For this interactome, we considered undirected edges; otherwise, the number of shortest paths is very low.
(TIFF)

Figure S11 **Shortest path distribution.** Contextualized in the PPIN interactome, for the subnetwork generated by the differentially expressed genes after viral infection (A, B, C, D, E, F, and G), and by lists of randomly selected genes (H).
(TIFF)

Figure S12 **Betweeness of PPIN interactome.** (A) Average betweenness, contextualized in the PPIN interactome, for the differentially expressed genes after viral infection and the whole interactome. NS denotes non-significant value according to a Mann-Whitney *U*-test. (B) Scatter plot of betweenness centrality and connectivity for the whole PPIN interactome. *Inter* corresponds to the betweenness computed to the PPIN interactome.
(TIFF)

Figure S13 **Protein-protein interaction network of** ***Potyviruses*** **inferred from empirical data gathered by different authors using the yeast two-hybrid system.** The parameters describing the network are: clustering coefficient 0.8713, network diameter 2, shortest path 110, characteristic path length 1.345, average number of neighbors 6.545, number of edges 45, network density 0.655, and number of self-loops 9. The 11 potyviral proteins are: P1 (trypsine-like serine proteinase), HC-Pro (helper-component during aphid transmission, RNA-silencing suppressor, and papain-like cystein proteinase), P3 (pathogenicity determinant), P3N-PIPO (movement protein), 6K1 (unknown function), CI (ATPase/RNA helicase and cell-to-cell movement), 6K2 (anchoring replication complexes to membranes), NIa-VPg (5′-linked protein involved in genome replication), NIa-Pro (trypsin-like serine proteinase), NIb (replicase), and CP (coat protein).
(TIFF)

Figure S14 **Clustering coefficient (** ***C*** **) for the subnetworks generated by the differentially expressed genes from several viral infections, contextualized in different** ***A. thaliana*** **interactomes.** *Rand* indicates the average value of randomly selected gene lists (100 replicates). NS denotes non-significant value following a one-tailed *z*-test. Horizontal dashed lines represent the cutoff value for statistical significance.
(TIFF)

Figure S15 **Number of connected components (** ***CC*** **) for the subnetworks generated by the differentially expressed genes from several viral infections.** Contextualized in *A. thaliana* (A) TRN, (B) TRN2, (C) PPIN, and (D) GGIN interactome. *Rand* indicates the average value of randomly selected gene lists (100 replicates).
(TIFF)

Figure S16 **Modularity coefficient (** ***M*** **) for the subnetworks generated by the differentially expressed genes from several viral infections.** Contextualized in different *A. thaliana* interactomes. *Rand* indicates the average value of randomly selected gene lists (100 replicates). NS denotes non-significant value following a one-tailed *z*-test. Horizontal dashed lines represent the cutoff value for statistical significance.
(TIFF)

Table S1 Assortativity coefficients (*A*) for the virus-associated subnetworks and the interactomes TRNTF (only considering transcription factors) and PPIN.
(DOCX)

File S1 **Differentially expressed genes (up/down) for all viral infections and those genes shared by pairs of viruses. In addition, we provide the similarity matrix.**
(XLS)

File S2 **Over-represented GO terms (up/down) for all viral infections and those GO terms shared by pairs of viruses. In addition, we provide the similarity matrix.**
(XLS)

File S3 **List of genetic interactions for the different interactomes we considered in this work (TRN, TRN2, PPIN, and GGIN).**
(XLS)
