# Supplementary figures and images for: A Meta-Analysis Reveals the Commonalities and Differences in Arabidopsis thaliana Response to Different Viral Pathogens
Source: PLoS One. 2012 Jul 12;7(7):e40526. doi: 10.1371/journal.pone.0040526 (PMC3395709; doi:10.1371/journal.pone.0040526)

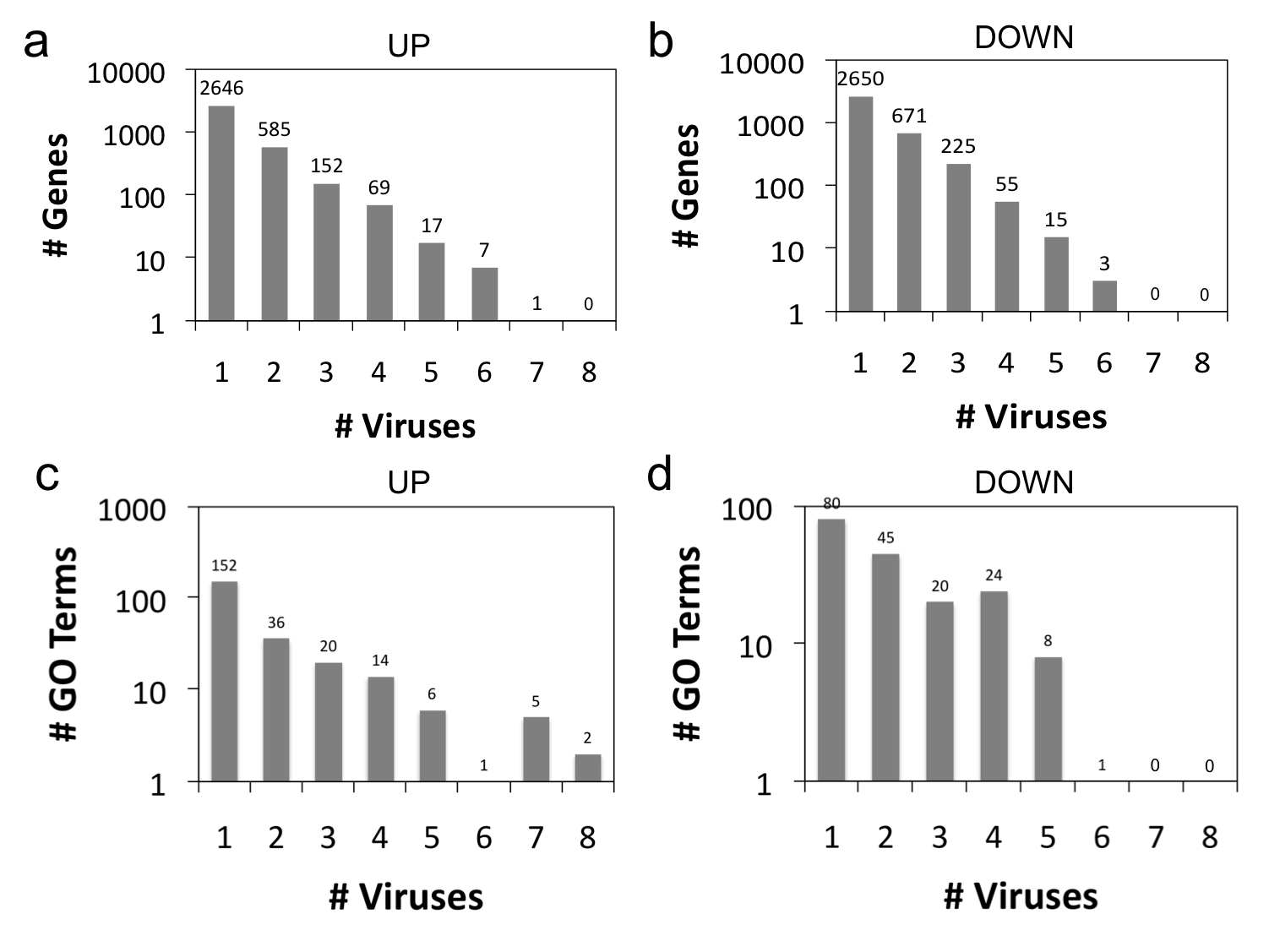

Supplement: Figure S1 — Altered gene expressions and GO terms. Distribution of genes up/down-expressed (A and B) and GO terms over/under represented (C and D) in A. thaliana after infection with the number of viruses indicated in the ordinates axis. The distributions are the result of comparing the differential patterns a posteriori between several viruses. (TIFF) [file pone.0040526.s001.tiff]

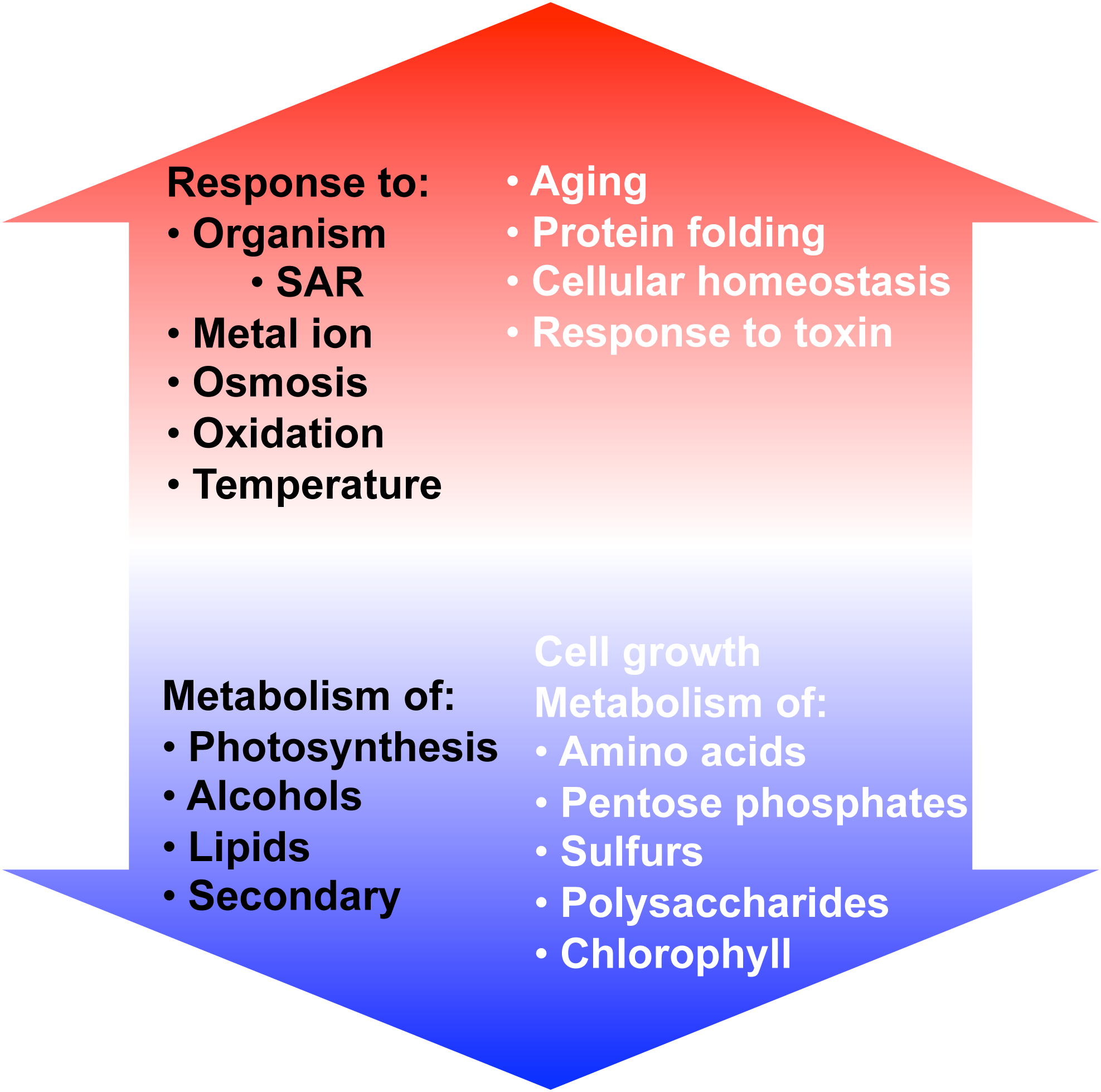

Supplement: Figure S2 — Altered VRFs. Summary of (red) over- and (blue) under-expressed VRFs representing biological processes. In black, consensus of VRFs for any viral infection (unspecific viral response). In white, consensus of VRFs specifically altered by Brassica-infecting viruses. (TIF) [file pone.0040526.s002.tif]

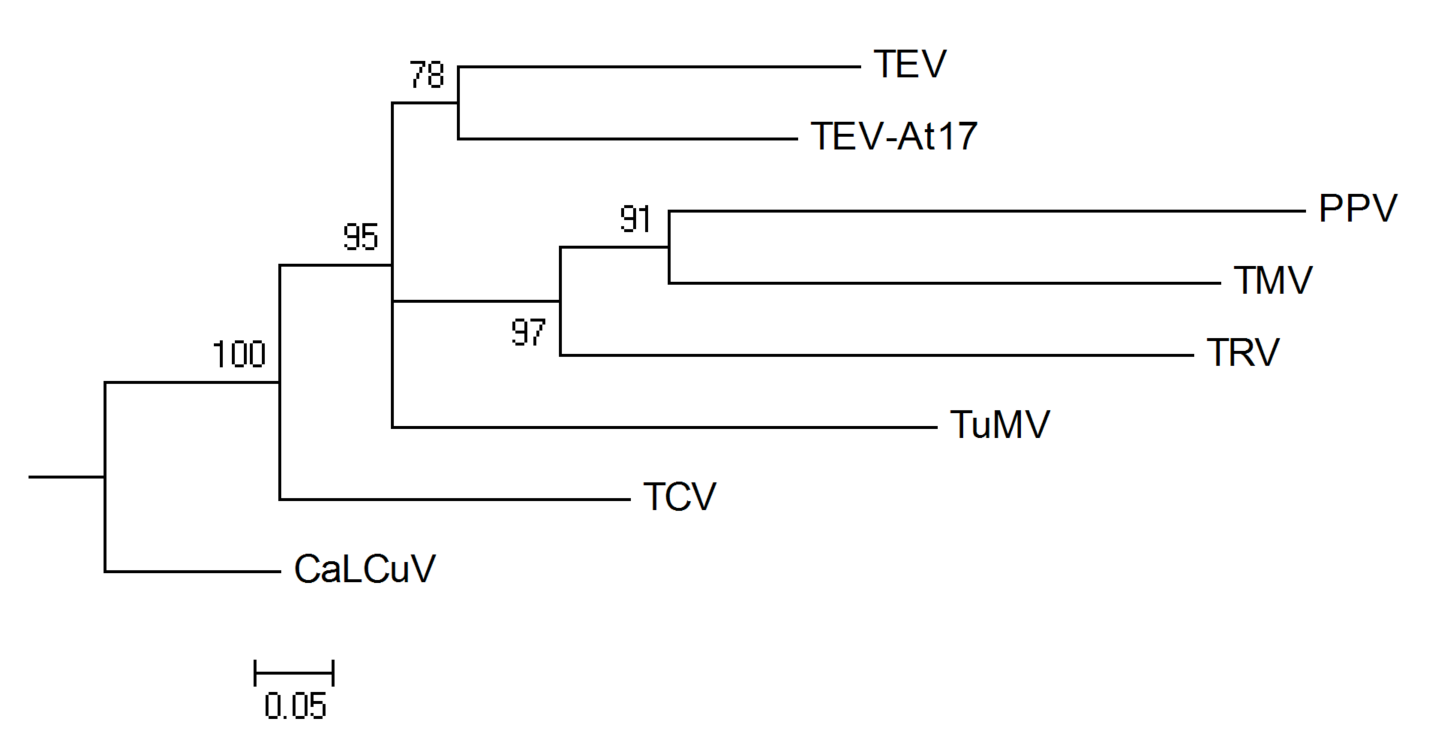

Supplement: Figure S3 — Neighbor-joining dendrogram constructed using the similarity matrix computed using the lists of over-represented GO terms. (TIF) [file pone.0040526.s003.tif]

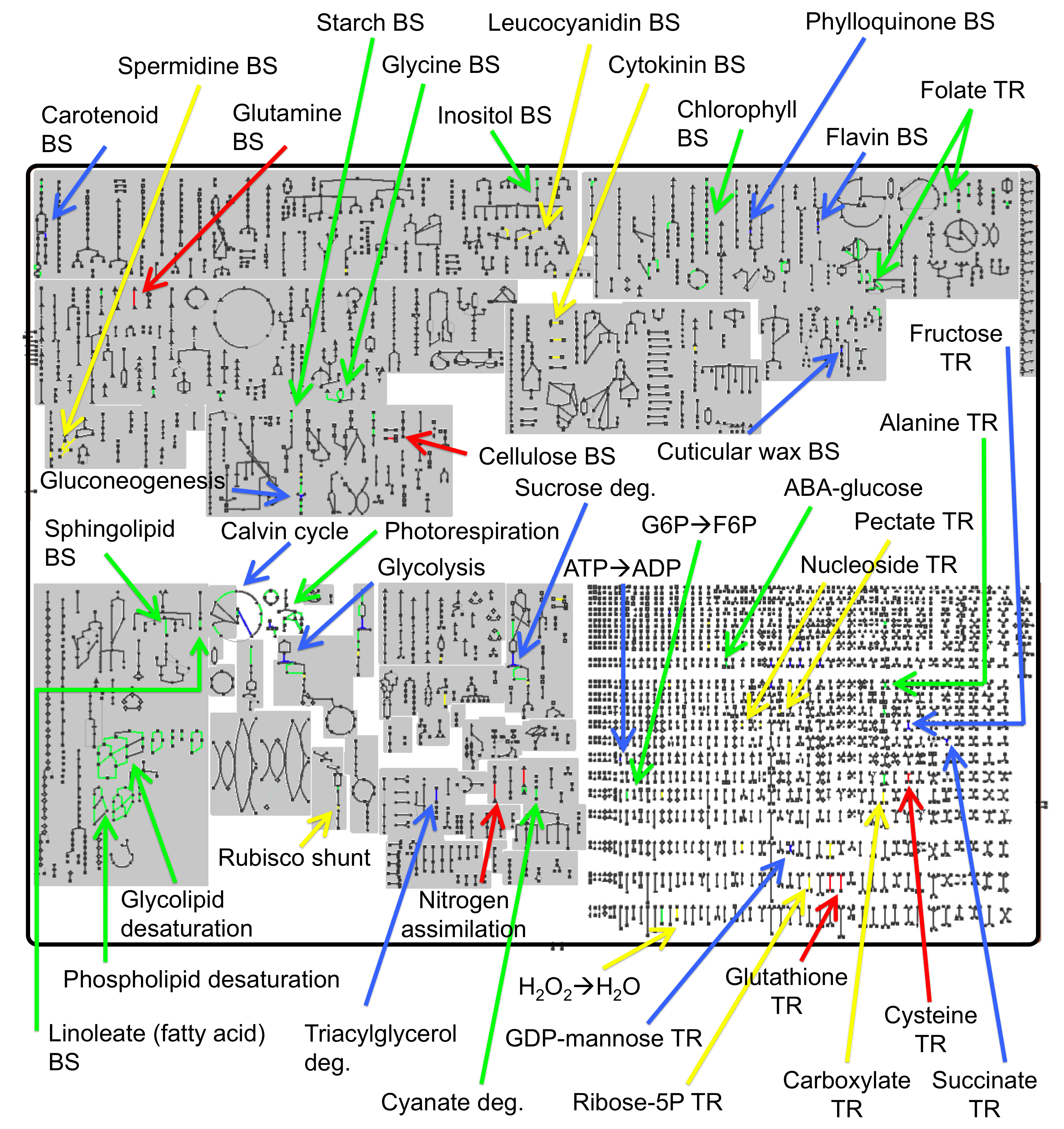

Supplement: Figure S4 — Metabolic map of A. thaliana. Highlighted the reactions altered by the unspecific viral response (VRGs in at least five of the total eight viral infections) and the specific Brassica-infecting virus response (VRGs in at least three of the four infections by Brassica-infecting viruses). Red (unspecific) and yellow (specific to Brassica-infecting viruses) reactions are over-expressed, whereas blue (unspecific) and green (Brassica-infecting) reactions are under-expressed. BS means biosynthesis, and TR, transformations. (TIFF) [file pone.0040526.s004.tiff]

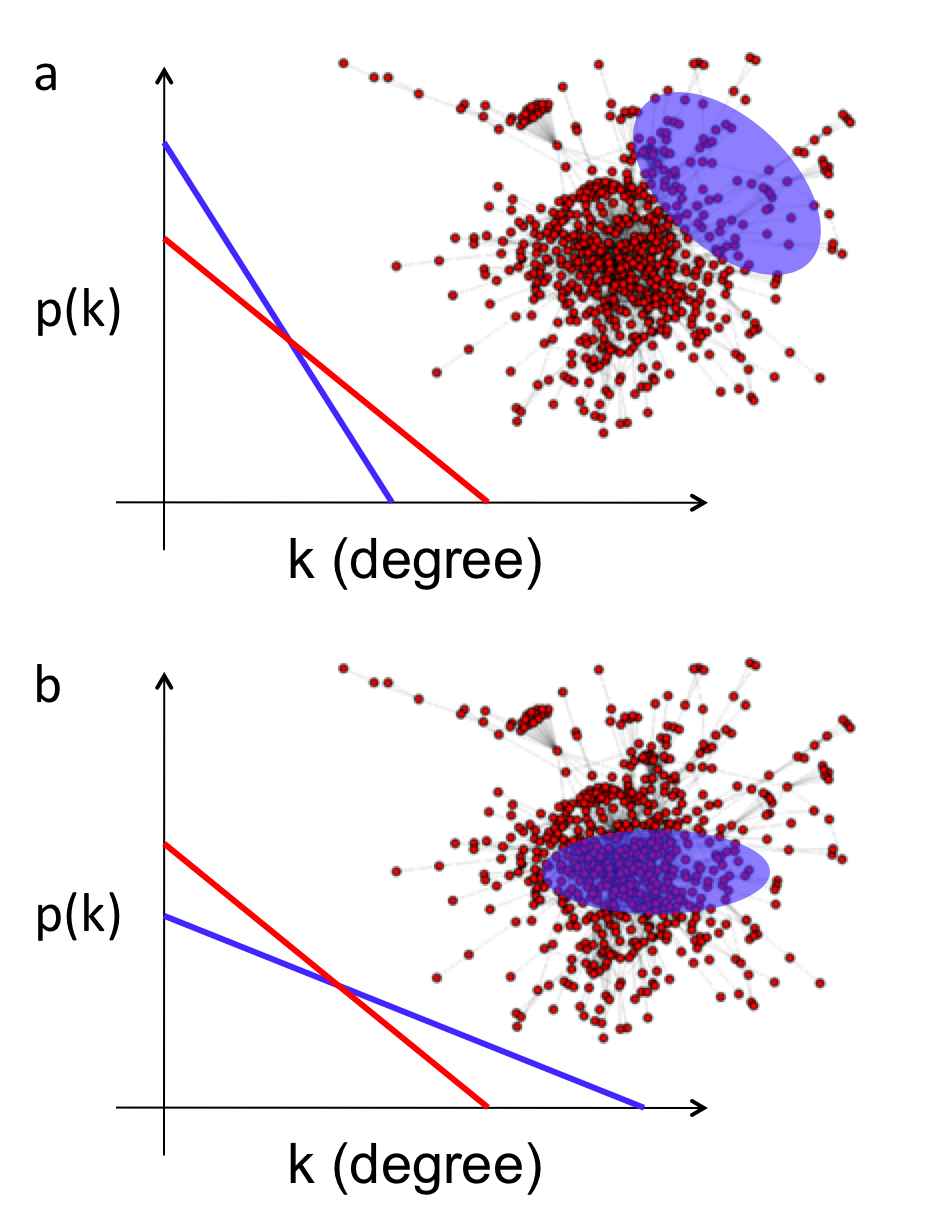

Supplement: Figure S5 — Predicted connectivity distribution for the whole plant interactome (red line) and the subnetwork generated by the total differentially (up/down) expressed VRGs (blue line). The set of VRGs can be in the periphery of the interactome if they have low connectivity (a) or in the core in they are highly connected (b). (TIFF) [file pone.0040526.s005.tiff]

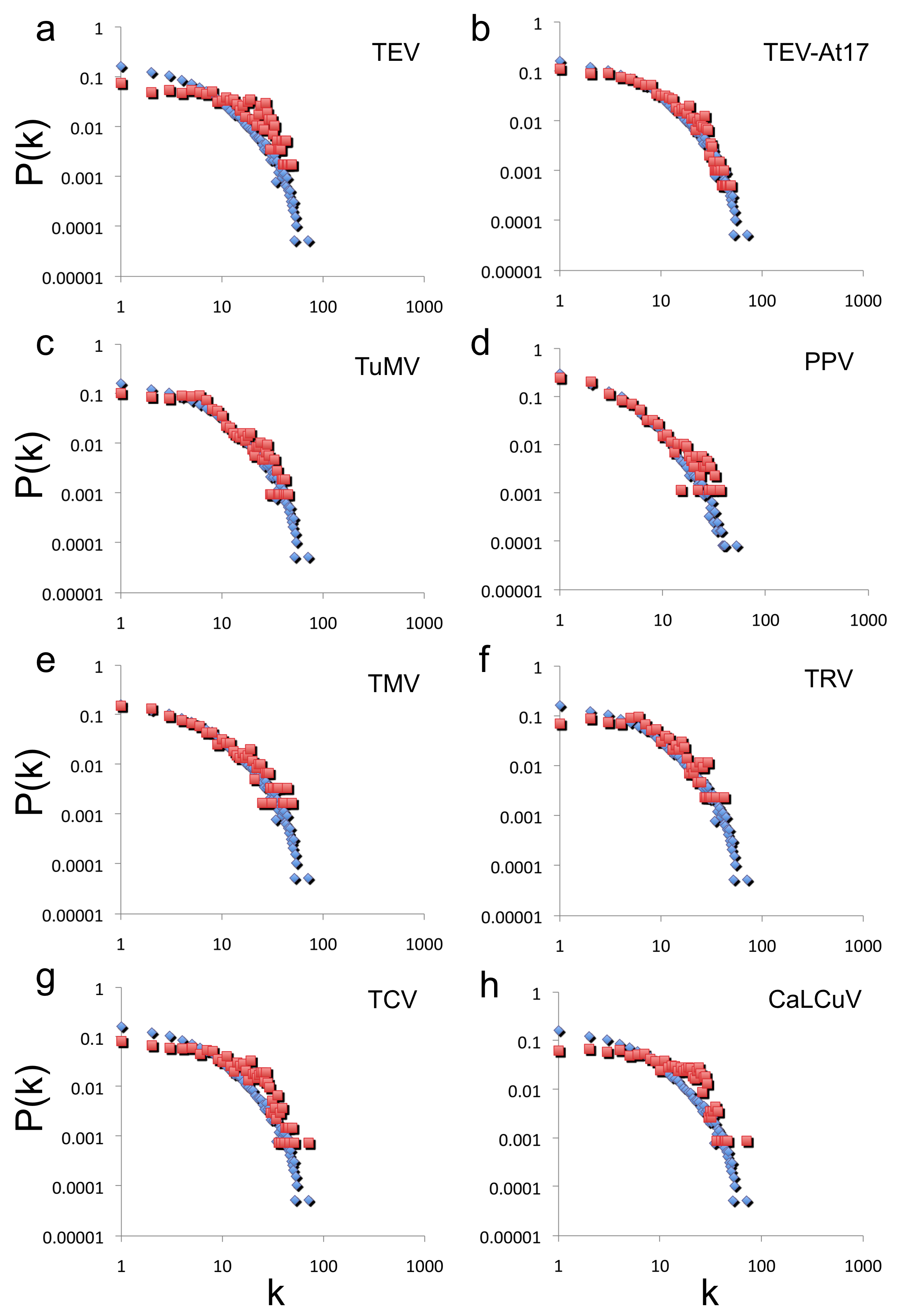

Supplement: Figure S6 — Incoming connectivity distribution. The distribution is contextualized in the TRN interactome, for the VRGs (red), and the whole interactome (blue). (TIFF) [file pone.0040526.s006.tiff]

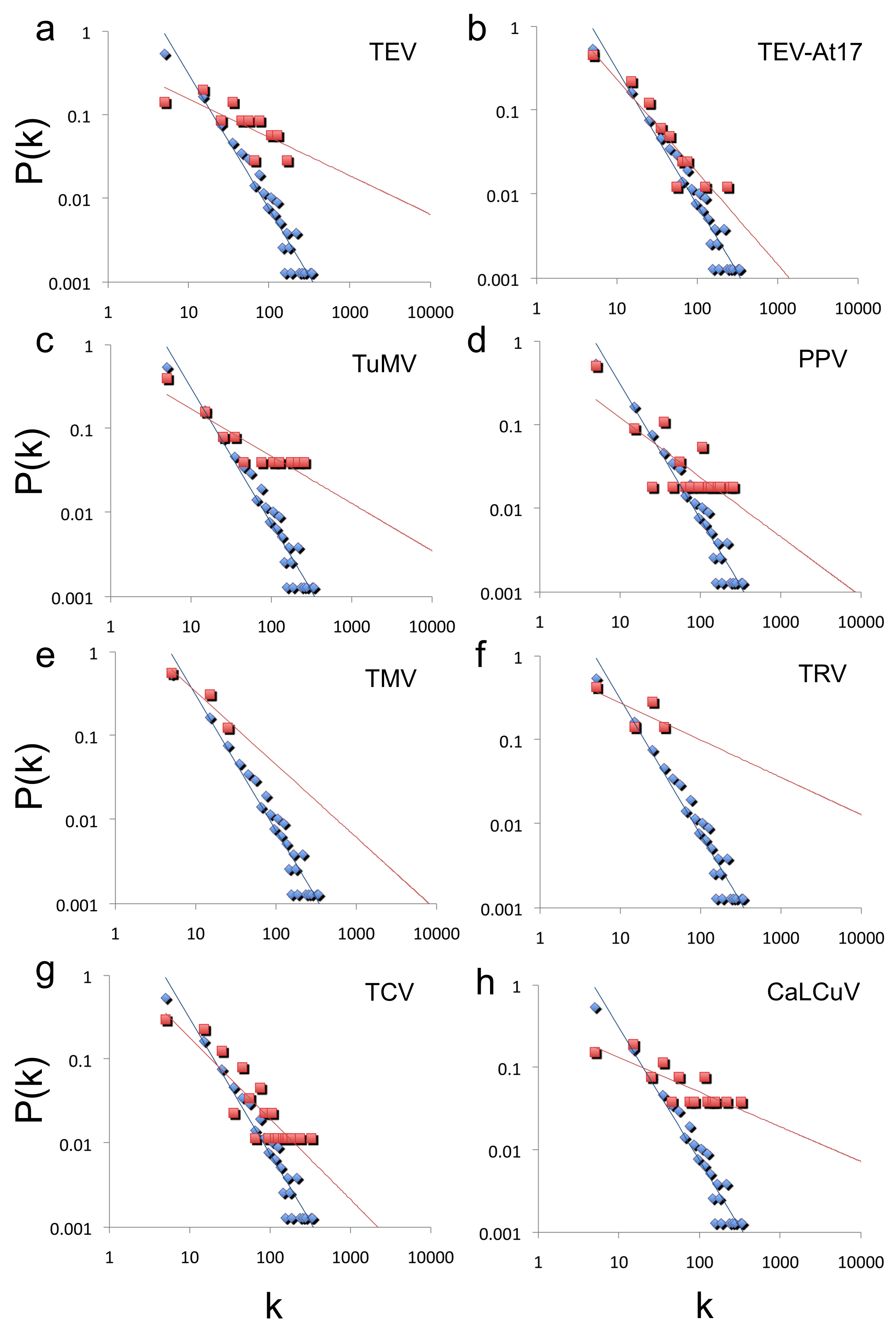

Supplement: Figure S7 — Outgoing connectivity distribution. The distribution is contextualized in the TRN2 interactome, for the VRGs (red), and the whole interactome (blue). (TIFF) [file pone.0040526.s007.tiff]

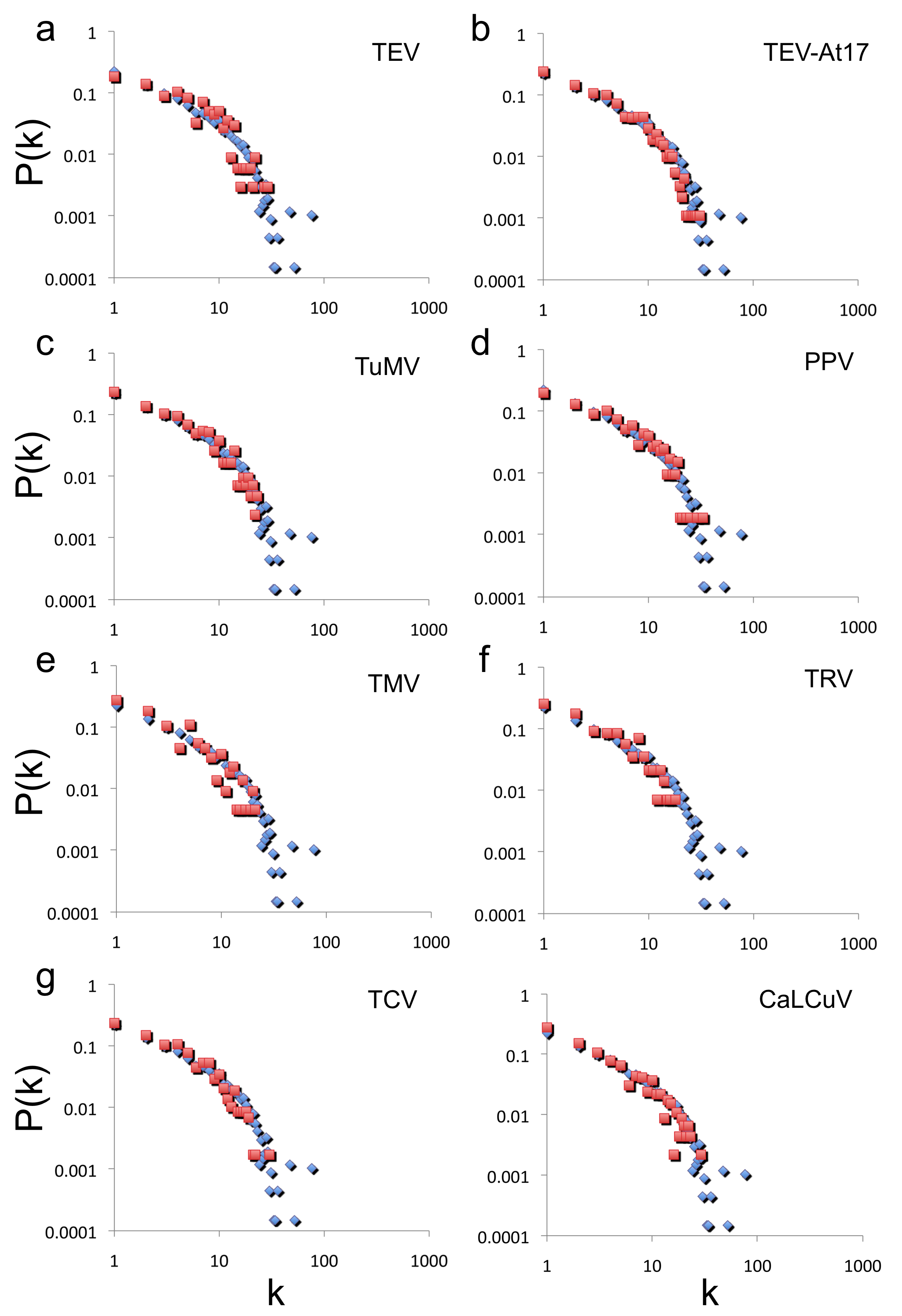

Supplement: Figure S8 — Connectivity distribution. The distribution is contextualized in the GGIN interactome, for the VRGs (red), and the whole interactome (blue). (TIFF) [file pone.0040526.s008.tiff]

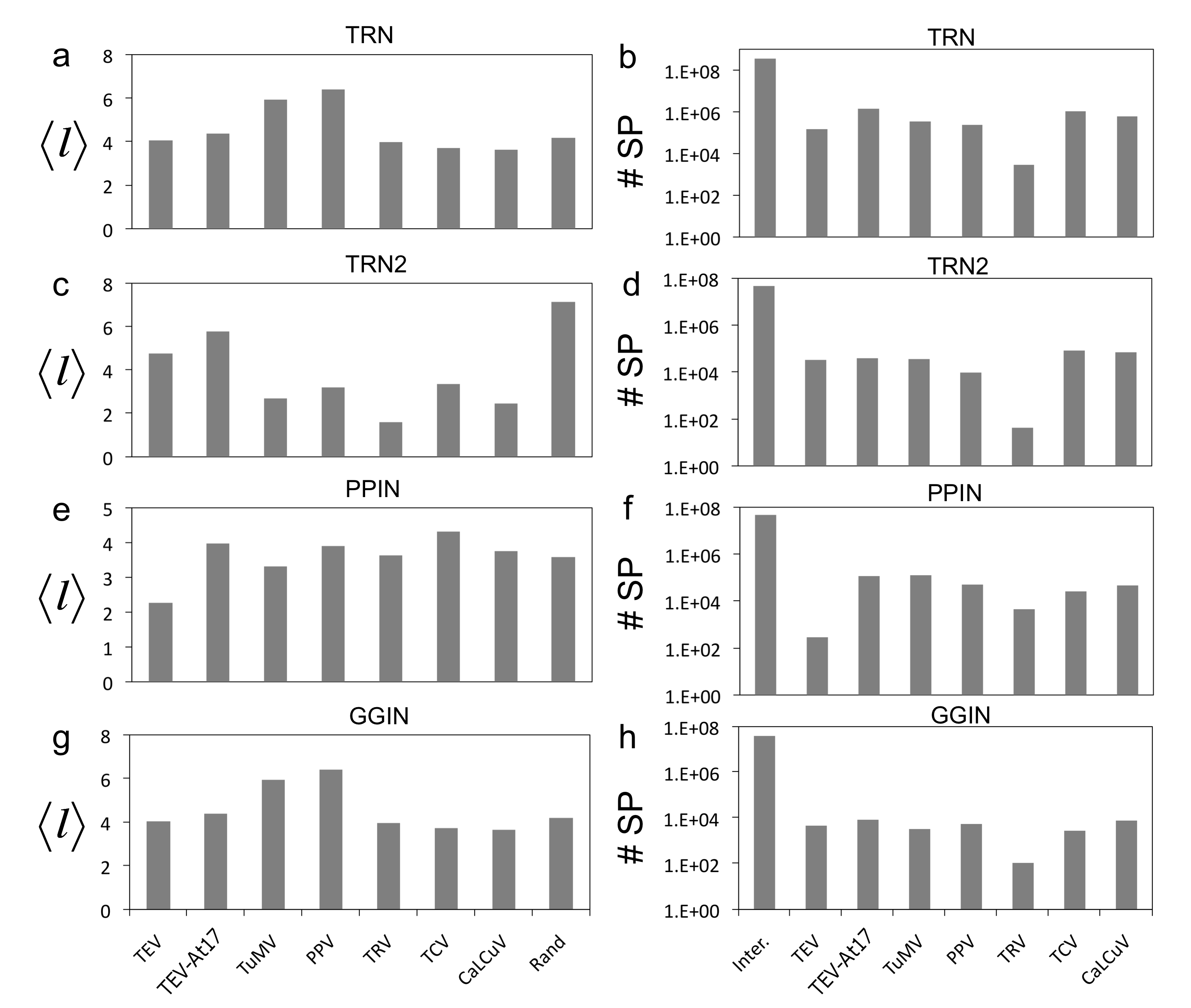

Supplement: Figure S9 — Statistics for the shortest paths. Shortest path average (A, C, E, and G) and total number of shortest paths (B, D, F, and H) for the subnetworks generated by the differentially expressed genes from several viral infections contextualized in different interactomes. Rand indicates the average value of randomly selected gene lists. Inter stands for interactome. For the TRN and TRN2 interactomes, we considered undirected edges; otherwise, the number of shortest paths is very low. (TIFF) [file pone.0040526.s009.tiff]

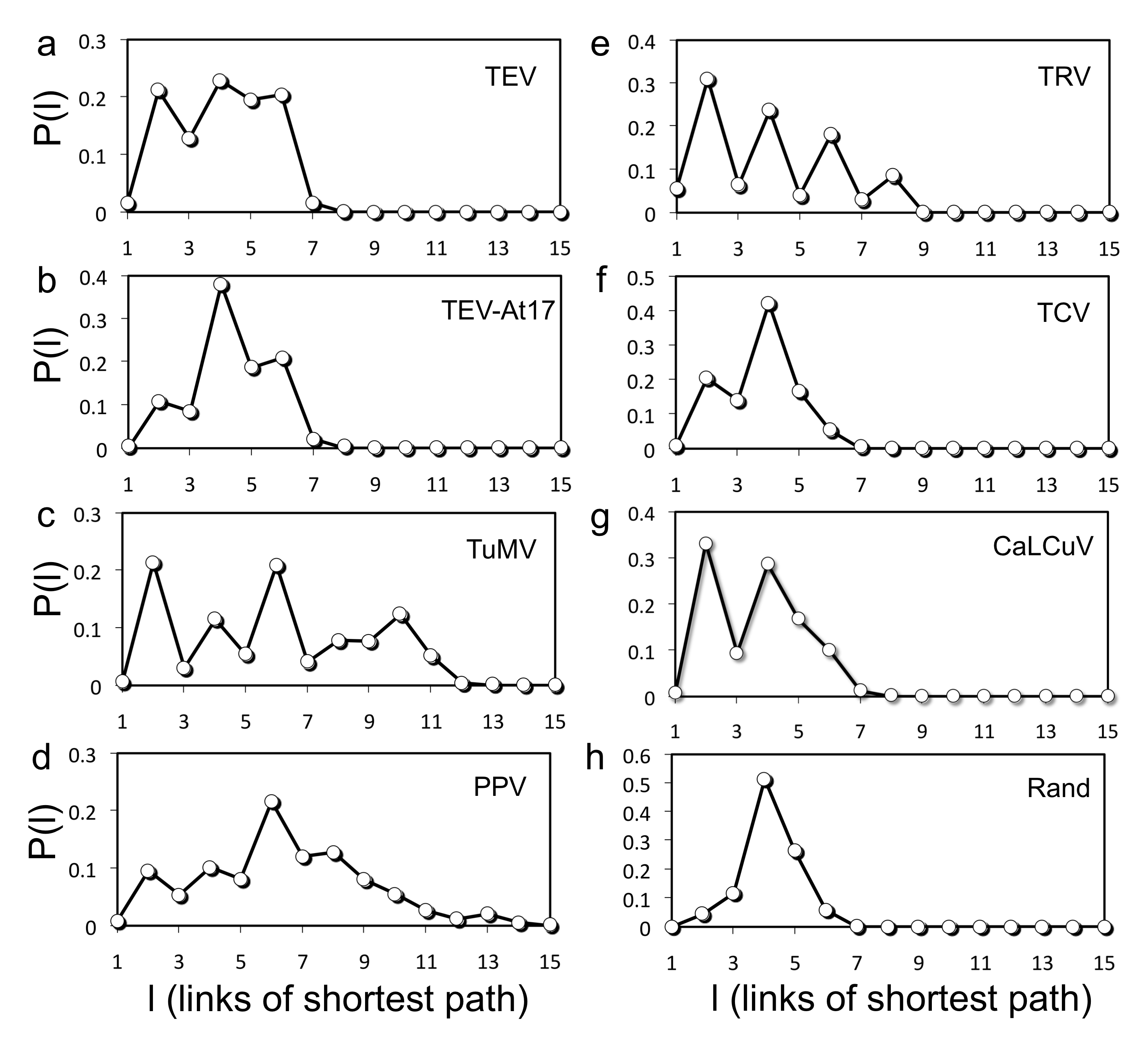

Supplement: Figure S10 — Shortest path distribution. Contextualized in the TRN interactome, for the subnetwork generated by the differentially expressed genes after viral infection (A, B, C, D, E, F, and G), and by lists of randomly selected genes (H). For this interactome, we considered undirected edges; otherwise, the number of shortest paths is very low. (TIFF) [file pone.0040526.s010.tiff]

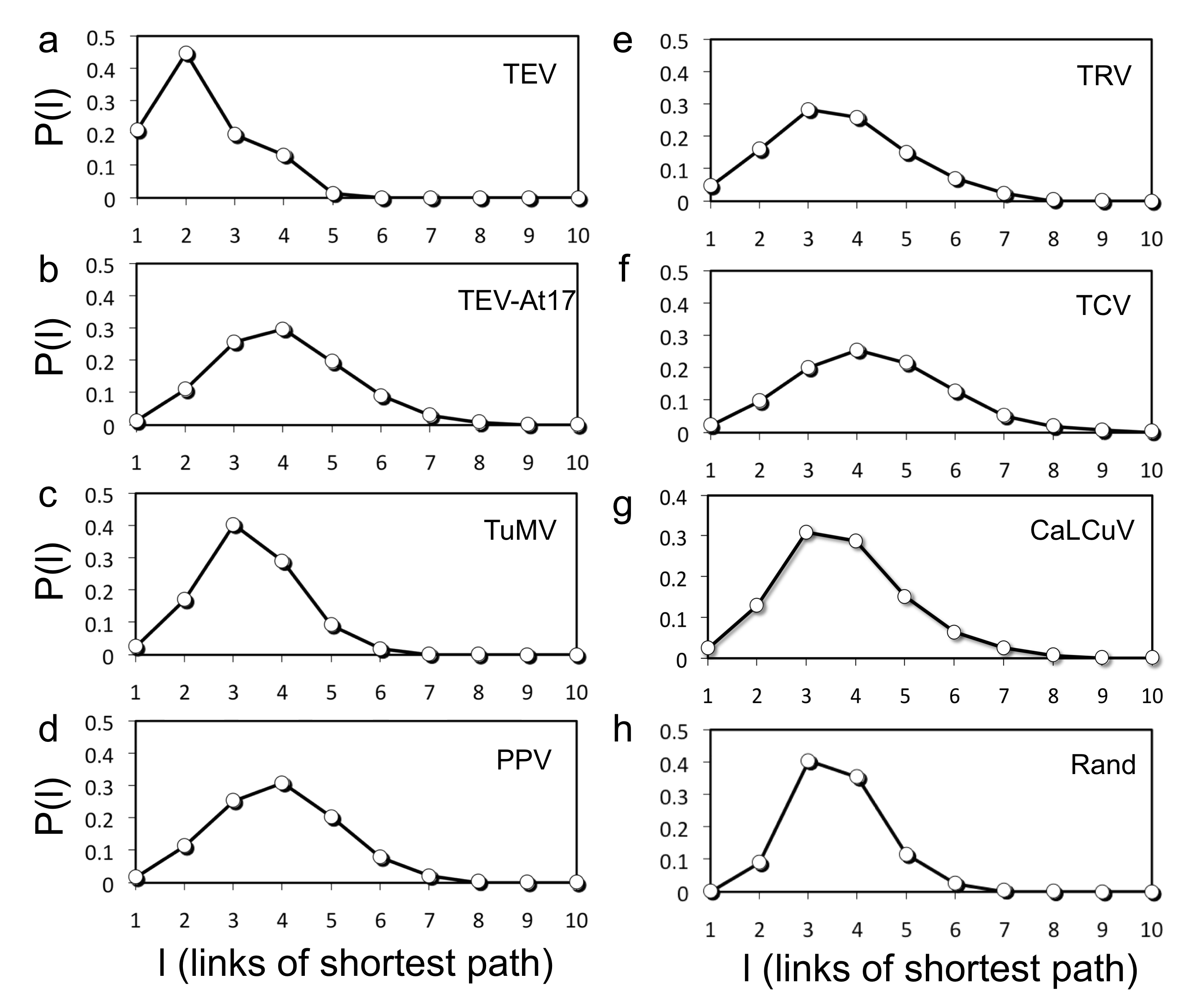

Supplement: Figure S11 — Shortest path distribution. Contextualized in the PPIN interactome, for the subnetwork generated by the differentially expressed genes after viral infection (A, B, C, D, E, F, and G), and by lists of randomly selected genes (H). (TIFF) [file pone.0040526.s011.tiff]

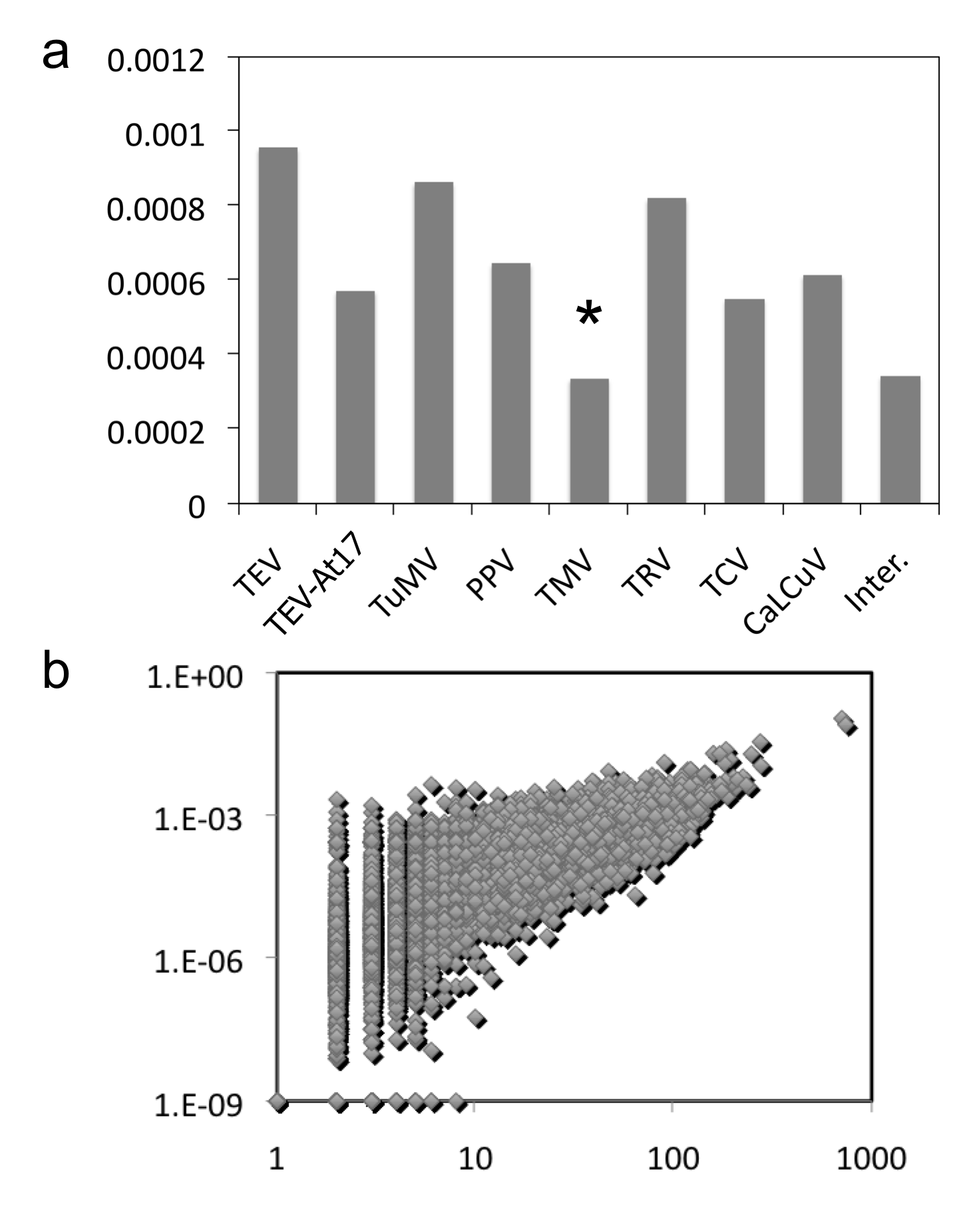

Supplement: Figure S12 — Betweeness of PPIN interactome. (A) Average betweenness, contextualized in the PPIN interactome, for the differentially expressed genes after viral infection and the whole interactome. NS denotes non-significant value according to a Mann-Whitney U-test. (B) Scatter plot of betweenness centrality and connectivity for the whole PPIN interactome. Inter corresponds to the betweenness computed to the PPIN interactome. (TIFF) [file pone.0040526.s012.tiff]

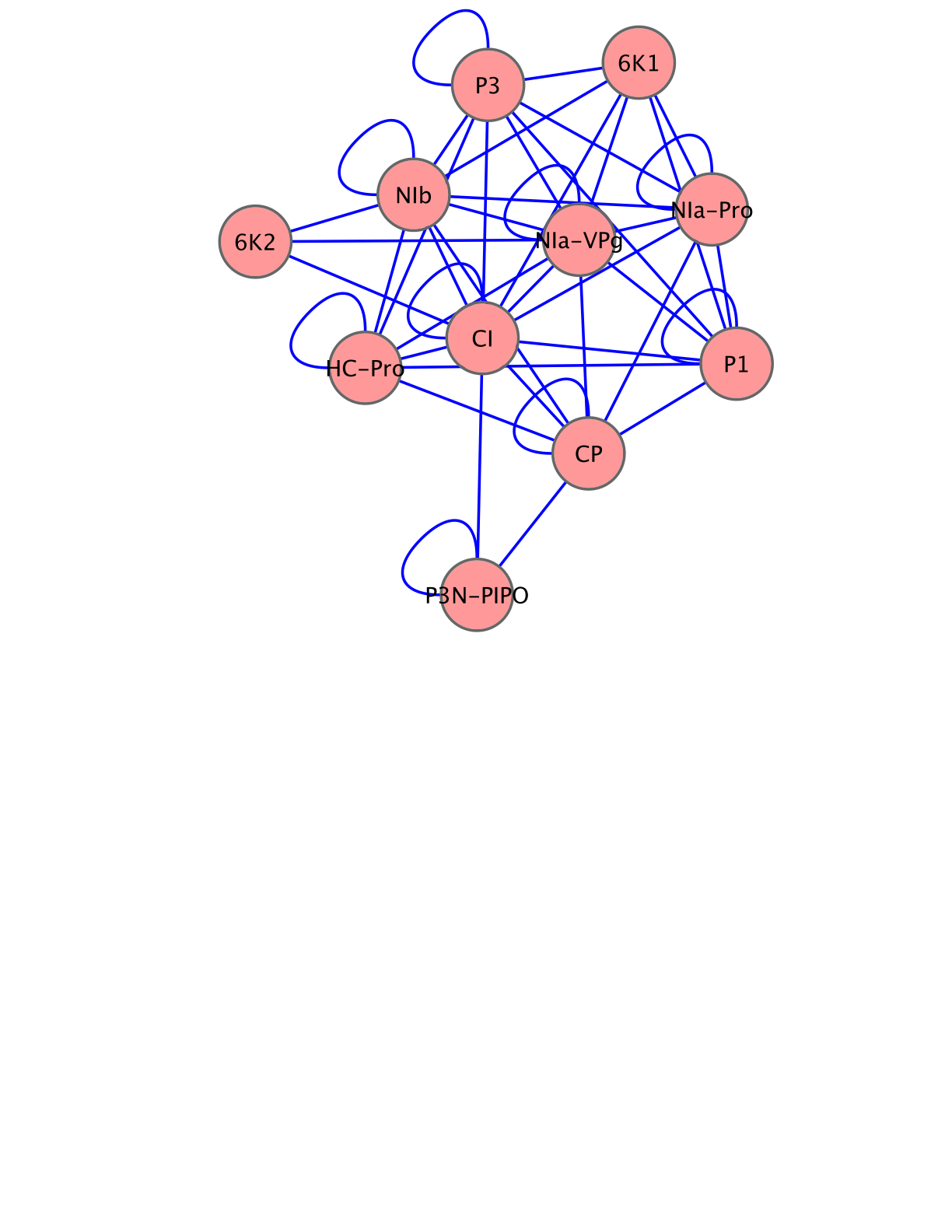

Supplement: Figure S13 — Protein-protein interaction network of Potyviruses inferred from empirical data gathered by different authors using the yeast two-hybrid system. The parameters describing the network are: clustering coefficient 0.8713, network diameter 2, shortest path 110, characteristic path length 1.345, average number of neighbors 6.545, number of edges 45, network density 0.655, and number of self-loops 9. The 11 potyviral proteins are: P1 (trypsine-like serine proteinase), HC-Pro (helper-component during aphid transmission, RNA-silencing suppressor, and papain-like cystein proteinase), P3 (pathogenicity determinant), P3N-PIPO (movement protein), 6K1 (unknown function), CI (ATPase/RNA helicase and cell-to-cell movement), 6K2 (anchoring replication complexes to membranes), NIa-VPg (5′-linked protein involved in genome replication), NIa-Pro (trypsin-like serine proteinase), NIb (replicase), and CP (coat protein). (TIFF) [file pone.0040526.s013.tiff]

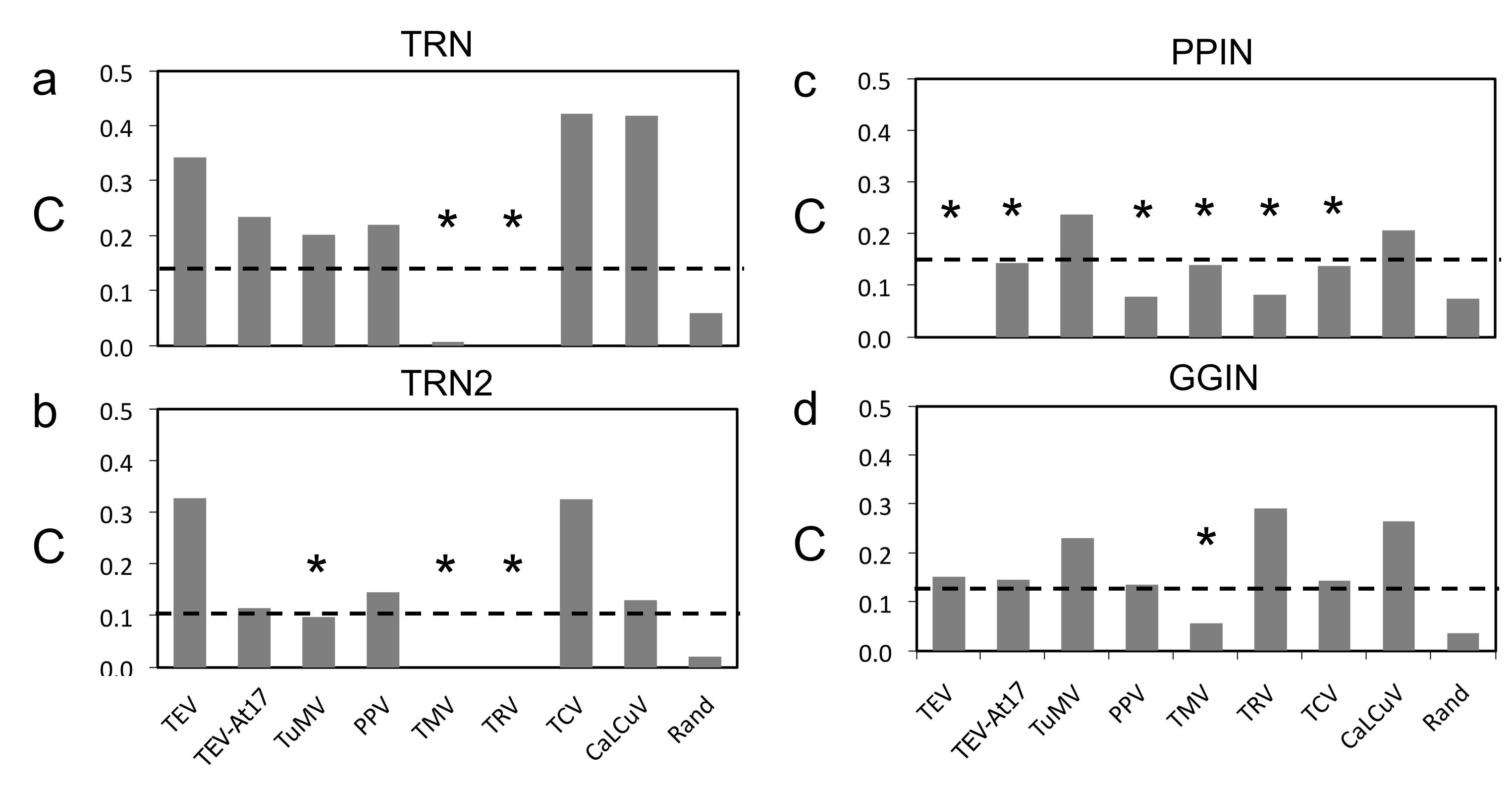

Supplement: Figure S14 — Clustering coefficient ( C ) for the subnetworks generated by the differentially expressed genes from several viral infections, contextualized in different A. thaliana interactomes. Rand indicates the average value of randomly selected gene lists (100 replicates). NS denotes non-significant value following a one-tailed z-test. Horizontal dashed lines represent the cutoff value for statistical significance. (TIFF) [file pone.0040526.s014.tiff]

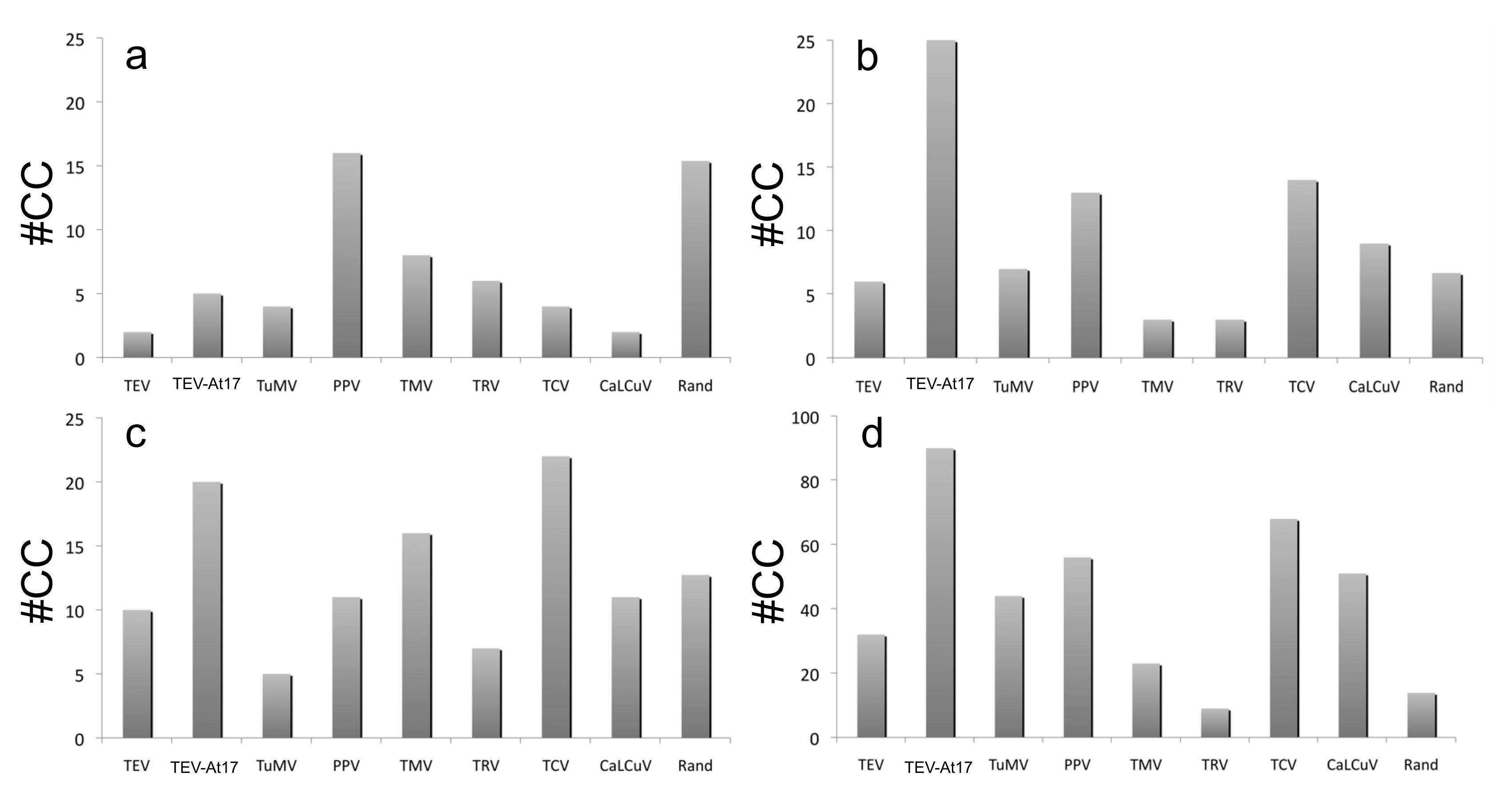

Supplement: Figure S15 — Number of connected components ( CC ) for the subnetworks generated by the differentially expressed genes from several viral infections. Contextualized in A. thaliana (A) TRN, (B) TRN2, (C) PPIN, and (D) GGIN interactome. Rand indicates the average value of randomly selected gene lists (100 replicates). (TIFF) [file pone.0040526.s015.tiff]

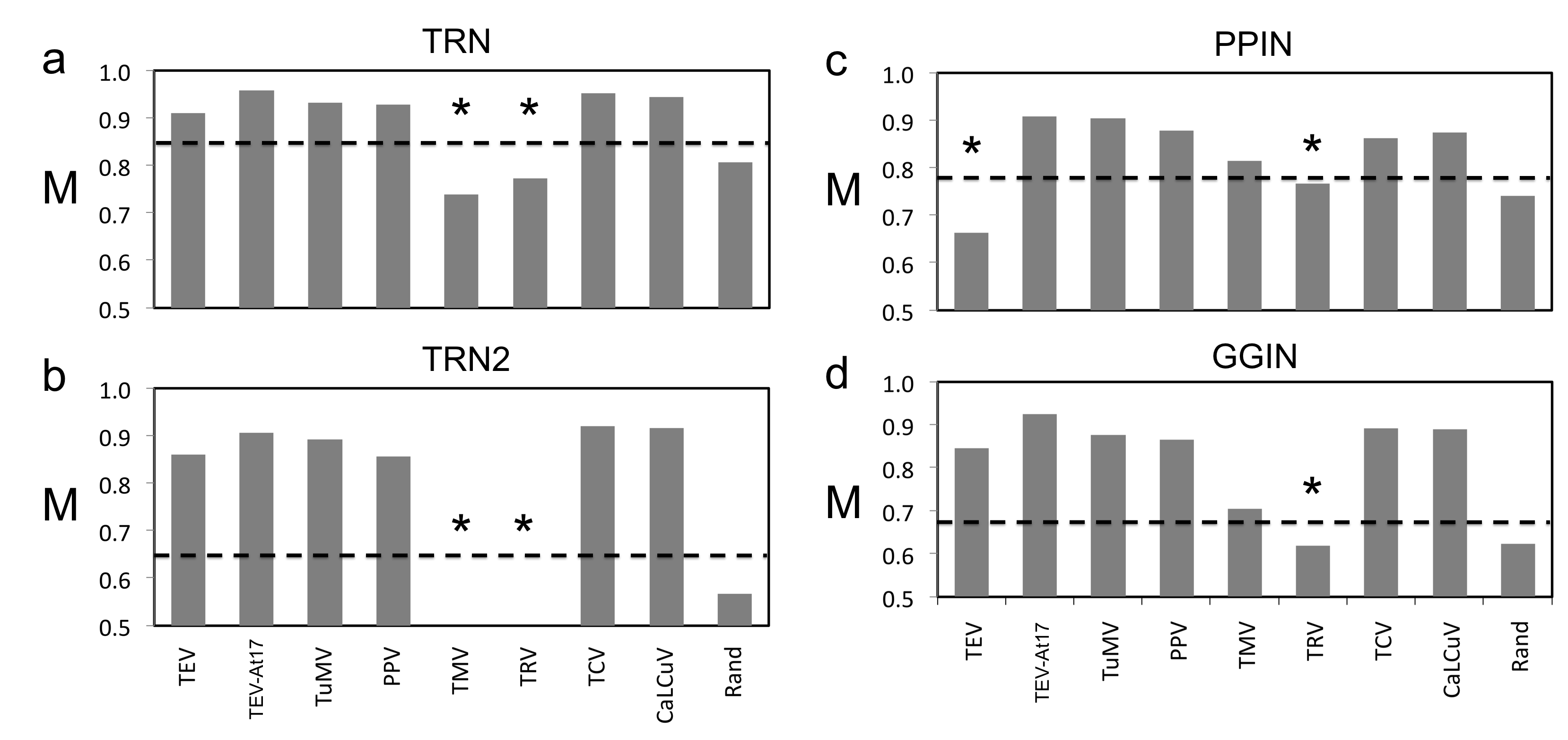

Supplement: Figure S16 — Modularity coefficient ( M ) for the subnetworks generated by the differentially expressed genes from several viral infections. Contextualized in different A. thaliana interactomes. Rand indicates the average value of randomly selected gene lists (100 replicates). NS denotes non-significant value following a one-tailed z-test. Horizontal dashed lines represent the cutoff value for statistical significance. (TIFF) [file pone.0040526.s016.tiff]
