# Supplementary material for: A Meta-Analysis Reveals the Commonalities and Differences in Arabidopsis thaliana Response to Different Viral Pathogens
Source: PLoS One. 2012 Jul 12;7(7):e40526. doi: 10.1371/journal.pone.0040526 (PMC3395709; doi:10.1371/journal.pone.0040526)
Supplement: Table S1 — Assortativity coefficients (A) for the virus-associated subnetworks and the interactomes TRNTF (only considering transcription factors) and PPIN. (DOCX) [file pone.0040526.s017.docx]

Table S1: Assortativity coefficients (*A*) for the virus-associated subnetworks and the interactomes TRN_TF_ (only considering transcription factors) and PPIN.

|  | *A* (*P*-value) in TRN_TF_ | *A* (*P*-value) in PPIN |
| --- | --- | --- |
| TEV | 0.2914 (0.0002) | -0.6163 (0.0341) |
| TEV-*At*17 | 0.1343 (< 0.0001) | -0.2783 (0.0008) |
| TuMV | 0.0700 (0.1287) | 0.1058 (0.0397) |
| PPV | 0.1810 (0.0101) | -0.2185 (0.0008) |
| TMV | 0.1200 (0.3188) | -1.6168 (0.0004) |
| TRV | -0.5422 (0.0040) | -0.0843 (0.4485) |
| TCV | 0.1929 (0.0012) | -0.2765 (0.0199) |
| CaLCuV | 0.1591 (0.0225) | -0.0122 (0.8858) |
| Interactome | 0.1692 (0.0022) | -0.0140 (0.3012) |
